# Dissection of the Functional Mechanism of Human Gut Bacterial Strain AD16 by Secondary Metabolites’ Identification, Network Pharmacology, and Experimental Validation

**DOI:** 10.3389/fphar.2021.706220

**Published:** 2021-11-05

**Authors:** Qin Wang, Yao Wang, Ya-Jing Wang, Nan Ma, Yu-Jie Zhou, He Zhuang, Xing-Hua Zhang, Chang Li, Yue-Hu Pei, Shu-Lin Liu

**Affiliations:** ^1^ Department of Medicinal Chemistry and Natural Medicine Chemistry, College of Pharmacy, Harbin Medical University, Harbin, China; ^2^ Genomics Research Center (State-Province Key Laboratories of Biomedicine-Pharmaceutics of China), College of Pharmacy, Harbin Medical University, Harbin, China; ^3^ Department of Microbiology, Immunology and Infectious Diseases, University of Calgary, Calgary, AB, Canada

**Keywords:** gut bacterial, secondary metabolites, network pharmacology, anticancer, AD16

## Abstract

Gut microbiota plays important roles in several metabolic processes, such as appetite and food intake and absorption of nutrients from the gut. It is also of great importance in the maintenance of the health of the host. However, much remains unknown about the functional mechanisms of human gut microbiota itself. Here, we report the identification of one anticancer gut bacterial strain AD16, which exhibited potent suppressive effects on a broad range of solid and blood malignancies. The secondary metabolites of the strain were isolated and characterized by a bioactivity-guided isolation strategy. Five new compounds, streptonaphthalenes A and B (1-2), pestaloficins F and G (3-4), and eudesmanetetraiol A (5), together with nine previously known compounds, were isolated from the effective fractions of AD16. Structures of the new compounds were established by 1D and 2D NMR and MS analysis, and the absolute configurations were determined by the CD method. The analysis of network pharmacology suggested that 3, 2, and 13 could be the key components for the anti-NSCLC activity of AD16. In addition to the PI3K–Akt signaling pathway, the proteoglycans in cancer pathway could be involved in the anti-NSCLC action of AD16.

## Introduction

The human gut microbiota is composed of an enormous diversity of microorganisms, including bacteria, fungi, and other microbes, which together play important roles in maintaining the dynamic homeostatic and healthy micro-environment of the host (Johnson et al., 2016; Thomas et al., 2017; Liang et al., 2018). In recent years, numerous discoveries have been reported on the human gut bacteria affecting human health and diseases, such as cardiovascular diseases, inflammatory diseases, obesity, and especially cancer (Hasan et al., 2020; Moritz et al., 2020). There has been mounting evidence supporting the roles of the gut bacteria in response to cancer (Johnson et al., 2016; Li et al., 2019), such as producing anticancer metabolites (Zhou et al., 2017). Although several bioactive metabolites from animal gut bacteria have been reported, such as sannastatin (Yang et al., 2011), few therapeutic metabolites have been identified from human gut bacteria (Rahim et al., 2019).

Our previous research suggested that the composition of the gut microbiota in lung cancer patients was radically different from that of healthy individuals, which had a higher abundance of bacteria of phylum Actinobacteria compared to the lung cancer patients (Zhuang et al., 2019). This finding prompted us to isolate more Actinobacteria from the human gut (Zhou et al., 2017). Strain AD16 was determined to belong to the Actinobacteria genus *Streptomyces* and showed potent cytotoxic activities against several cancer cell lines both *in vitro* and *in vivo*. Based on the promising results, AD16 was selected for phytochemical studies with a focus on its secondary metabolites responsible for the observed anticancer properties. We identified five new compounds, including streptonaphthalenes A and B (1-2), pestaloficins F and G (3-4), and eudesmanetetraiol A (5), along with nine previously known ones, cyclo-(leucyl-histidyl) (6) (Furukawa et al., 2012), 4,10-dihydroxy-10-methyl-undec-2-en-1, 4-olide (**7**) (Cho et al., 2001), 4-acetyl-benzoxazolin-2-one (8) (Fielder et al., 1994), cinnamic acid (9) (Ai et al., 2010), indole-3-carboxylic acid (10) (Qian et al., 2014), 1-(1H-indol-3-yl)-ethanone (11) (Kamble et al., 2020), DBP (12) (Chang et al., 2013), 4-hydroxy-8-[6-hydroxy-1,3,7-trimethyl-2-oxo-oct-3-enyl]-5-methyl-oxocan-2-one (13) (Tapiolas et al., 1991), and 1,2,4-triazolenucleoside (14) (Zhou et al., 2010). We also applied network pharmacology analysis to investigate the underlying mechanisms of the anticancer effects of AD16.

In this paper, we describe the anticancer activities of gut bacterial strain AD16, the isolation and structural elucidation of five new compounds, along with nine known ones *via* bioactivity-guided isolation, and the network analysis of the compounds from AD16. The chemical structures of the isolated compounds were deduced by means of their physico-chemical properties, as well as the analysis of their spectroscopic data. This work demonstrated that gut microbiota is a rich source of potential cancer therapeutics for further studies and future clinical applications.

## Materials and Methods

### General Experimental Procedures

Optical rotations were measured on a Nicolet iS5 (Thermo, United States) spectrometer, and UV spectra were recorded on an Evolution 220 (Thermo, United States) UV/Vis spectrometer. IR spectra were obtained using a JASCO FT/IR-480 plus spectrometer. ^1^H-NMR and 2D NMR spectra were measured on a Bruker AV-600 spectrometer, while ^13^C-NMR spectra were measured on a Bruker AV-400 spectrometer. CD spectra were recorded on MOS 450 (Bio-Logic, France). HRESIMS data were determined by an Agilent Q-TOF 6520 mass spectrometer. Open column chromatography (CC) was performed using silica gel (200–300 mesh, Qingdao Haiyang Chemical Group Corp., Qingdao, China), ODS (50 μm, YMC, Japan), and HW-40 (Tosoh, Japan). Thin-layer chromatography (TLC) was performed using precoated silica gel plates (silica gel GF254, 1 mm, Yantai).

### Isolation and Identification of AD16

The detailed collection and isolation procedures of the bacteria from human fecal specimens were done as previously reported (Zhuang et al., 2019). A colony of bacteria that showed potent anticancer activities was identified as closely related to *Streptomyces* and was given the strain name AD16 (gene bank No. KU883604.1). This strain was isolated from the fecal specimen of a healthy girl (5 years old) and stocked in the Laboratory of Genomics Research Center of Harbin Medical University (Harbin, China). All the experiments of the study were consistent with standard biosecurity and institutional safety procedures. All microbes were handled in the BSL-2 laboratory.

### Cell Culture and CCK-8 Assay

Human solid cancer cell lines, including cervical cancer HeLa, ovarian cancer A2780, lung cancer A549, and colorectal cancer HCT116, were cultured in Dulbecco’s modified Eagle’s medium (DMEM) with 10% fetal bovine serum. Ovarian cancer cell lines ES-2 and OV-90 were cultured in McCoy’s 5A medium with 10% fetal bovine serum. All the cultures were maintained in an incubator at 37°C with 5% CO_2_ in a humidified atmosphere.

Cell viability was measured by the Cell Counting Kit (CCK)-8 (Dojindo, Tokyo, Japan) assay. A549 cells (5.0 × 10^3^ cells per well) were seeded into 96-well plates (Corning, NY) and cultured for 24 h. The cells were then incubated with fresh media containing the compounds under study at various concentrations for 24, 48, or 72 h. After incubation, the media were removed and the wells were washed twice with PBS to remove non-adherent cells. Then, 100 μL fresh medium and 10 μL CCK-8 were added to each well at the indicated time points. The cells were further incubated at 37°C for 60 min. The absorbance of the samples was measured at 492 nm using a Bio-Rad model 3550 microplate reader (Richmond, CA).

### Morphological Assessment

Morphological changes of cells treated with AD16 supernatant or metabolites were inspected by phase-contrast inverted microscopy (Zeiss Axiocam ERc 5s, Germany). The performance of the experiments and the determination of experimental results were completed blindly and separately by at least two different persons.

### Cell Apoptosis Analysis

The cells were incubated in the medium containing culture supernatant of AD16 for 6 h. The cells were harvested, washed twice with cold 1 × PBS, and re-suspended in 100 μL 1 × binding buffer at a density of 1 × 10^5^ cells/mL. The cells were then stained with 5 μL Annexin V and 5 μL PI (BD Biosciences) for 15 min in dark condition at room temperature. After staining, we added 400 µL of 1 × binding buffer to each tube. The samples were subjected to analysis by flow cytometry (BD FACSCanto^TM^ II). The early apoptosis was evaluated based on the percentage of Annexin V–positive and PI-negative cells, while the late apoptosis was evaluated based on the percentage of Annexin V–positive and PI-positive cells.

### Statistical Analysis

Statistical analysis was presented as the mean ± standard deviation (SD) of at least three independent experiments. Student’s t-test, chi-square test, and Spearman’s rank correlation analysis were used to assess the means of the different samples with SPSS statistical software version 17.0 and GraphPad Prism software. The statistical significance was accepted at *p* < 0.05. Our study closely followed the line of randomness and preciseness to ensure reproducibility.

### Fermentation, Extraction, and Isolation of AD16

Strain AD16 was inoculated in 500 ml conical flasks (497 bottles in total) containing 300 ml GRC1 medium (20 g of soluble starch, 1 g of KNO_3_, 0.5 g of KH_2_PO_4_, 0.5 g of MgSO_4_ 7H_2_O, and 0.5 g of NaCl in 1 L of distilled water) for 15 days at 150 rpm/min at room temperature. D101 macroporous resin was soaked with the whole culture for 24 h and then eluted with water and EtOH–H_2_O (95:5, V/V), respectively. The EtOH–H_2_O eluate was concentrated by a rotary evaporator in vacuum to afford 33.8 g of dry material. An aliquot (31.7 g) was applied to an ODS column (3.5*46 cm; 50 μm) and eluted with MeOH–H_2_O in gradient to give 13 fractions (K1–K13).

Fraction K4 [MeOH–H_2_O (20:80, V/V) eluate, 0.7 g] was subjected to HW-40 CC, eluted with MeOH–H_2_O in gradient, to yield 11 subfractions (K4A–K4K). Subfraction K4J [MeOH–H_2_O (100:0, V/V) eluate, 15.6 mg] was purified by preparative HPLC (Cosmosil C18, 5 μm, 20 × 250 mm, Cosmosil) with MeOH–H_2_O (15:85, V/V) to afford compound 6 (5.2 mg, t_R_ = 22.5 min).

Fraction K8 [MeOH–H_2_O (25:75–30:70, V/V) eluate, 0.9 g] was subjected to HW-40 CC, eluted with MeOH–H_2_O in gradient, to yield 11 subfractions (K8A–K8K). Subfraction K8D [MeOH–H_2_O (10:90, V/V) eluate, 158.8 mg] was subjected to silica-gel CC eluted with CH_2_Cl_2_–MeOH in gradient to yield nine subfractions (K8D1–K8D9). Subfraction K8D2 [CH_2_Cl_2_–MeOH (25:1, V/V) eluate, 49.9 mg] was subjected to Sephadex LH-20 CC eluted with MeOH to yield three subfractions (K8D2A–K8D2C). K8D2B (MeOH eluate, 9.3 mg) was purified by silica-gel CC with a cyclohexane–acetone gradient to yield four subfractions (K8D2B1–K8D2B4). After combining K8D2B2 [cyclohexane–acetone (7:2, V/V) eluate, 4.8 mg] and K8D2C (MeOH eluate, 4.8 mg) to the new fraction, it was further purified by preparative HPLC (Cosmosil C18, 5 μm, 10 × 250 mm, Cosmosil) with MeOH–H_2_O (18:82, V/V) to afford compound 4 (2.8 mg, t_R_ = 54 min). Fraction K8K [MeOH–H_2_O (50:50–100:0, V/V) eluate, 12.6 mg] was purified by Sephadex LH-20 CC eluted with MeOH to afford compound 10 (1.3 mg).

Fraction K9 [MeOH–H_2_O (30:70–50:50, V/V) eluate, 0.6 g] was subjected to HW-40 CC, eluted with MeOH–H_2_O in gradient, to yield 12 subfractions (K9A–K9L). Subfraction K9B [MeOH–H_2_O (15:85, V/V) eluate, 49.6 mg] was subjected to silica-gel CC with cyclohexane–acetone to yield three subfractions (K9B1–K9B3). The fine fraction K9B2 [cyclohexane–acetone (1:1, V/V) eluate, 10.0 mg] was purified by Sephadex LH-20 CC eluted with MeOH to afford compound 5 (3.0 mg). Fraction K9C [MeOH–H_2_O (30:70, V/V) eluate, 25.5 mg] was purified by preparative HPLC (Cosmosil C18, 5 μm, 10 × 250 mm, Cosmosil) with MeOH–H_2_O (20:80, V/V) to afford compound 14 (4.7 mg, t_R_ = 66.0 min). Fraction K9H [MeOH–H_2_O (30:70, V/V) eluate, 24.8 mg] was purified by Sephadex LH-20 CC eluted with MeOH to afford compound 8 (3.1 mg).

Fraction K10 [MeOH–H_2_O (50:50–70:30, V/V) eluate, 1.5 g] was subjected to HW-40 CC, eluted with MeOH–H_2_O in gradient, to yield 17 subfractions (K10A–K10Q). Subfraction K10J [MeOH–H_2_O (30:70, V/V) eluate, 18.9 mg] was subjected to silica-gel CC eluted with a CH_2_Cl_2_–MeOH gradient to afford compound 2 [CH_2_Cl_2_–MeOH (25:1, V/V) eluate, 2.0 mg]. Subfraction K10M [MeOH–H_2_O (50:50, V/V) eluate, 23.2 mg] was subjected to silica-gel CC with cyclohexane–acetone (7:1, V/V) to afford compound 11 (2.4 mg) and yield two subfractions (K10M1-K10M2). The fine fraction K10M1 [cyclohexane–acetone (7:1, V/V) eluate, 7 mg] was purified by preparative TLC with CH_2_Cl_2_–MeOH (20:1, V/V) to afford compound 9 (3.5 mg).

Fraction K11 [MeOH–H_2_O (70:30, V/V) eluate, 6.9 g] was subjected to HW-40 CC, eluted with MeOH–H_2_O in gradient, to yield nine subfractions (K11A–K11I). Subfraction K11C [MeOH–H_2_O (40:60, V/V) eluate, 1.3 g] was subjected to HW-40 CC eluted with an MeOH–H_2_O gradient to yield nine subfractions (K11C1–K11C9). Fraction K11C4 [MeOH–H_2_O (15:85, V/V) eluate, 159.5 mg] was subjected to Sephadex LH-20 CC eluted with MeOH to yield 10 subfractions (K11C4A–K11C4J). Subfraction K11C4C (MeOH eluate, 24.2 mg) was purified by preparative HPLC (Cosmosil C18, 5 μm, 10 × 250 mm, Cosmosil) with MeOH–H_2_O (48:52, V/V) to afford compound 13 (2.0 mg, t_R_ = 115.0 min). Subfraction K11C4D (MeOH eluate, 18.3 mg) was purified by preparative HPLC (Cosmosil C18, 5 μm, 10 × 250 mm, Cosmosil) with MeOH–H_2_O (48:52, V/V) to afford compound **7** (2.4 mg, t_R_ = 39.0 min). Subfraction K11C4F (MeOH eluate, 21.7 mg) was purified by preparative HPLC (Cosmosil C18, 5 μm, 20 × 250 mm, Cosmosil) with MeOH–H_2_O (43:57, V/V) to afford compound 3 (2.1 mg, t_R_ = 46.0 min). Subfraction K11C9 [MeOH–H_2_O (50:50, V/V) eluate, 82.4 mg] was subjected to silica-gel CC with a cyclohexane–acetone gradient to yield eight subfractions (K11C9A–K11C9H). The fine fraction K11C9D [cyclohexane–acetone (4:1, V/V) eluate, 3.4 mg] was purified by preparative TLC with cyclohexane–acetone (1:1, V/V) to afford compound 1 (1.8 mg). Fraction K11F [MeOH–H_2_O (60:40, V/V) eluate, 0.8 g] was purified by preparative HPLC (Cosmosil C18, 5 μm, 20 × 250 mm, Cosmosil) with MeOH–H_2_O (77:23, V/V) to afford compound 12 (22.1 mg, t_R_ = 40.0 min).

#### Streptonaphthalene A (1)

White amorphous solid; [α] ${\;}^{20}{\;}_{\text{D}}$ 98 (*c* 0.1, MeOH); UV (MeOH) λ_max_ (log) 230 (4.22) nm, 275 (3.94) nm; CD (MeOH) 230 (Δ*ε* −2.63), 296 (Δ*ε* −1.07) nm; IR 3354 cm^−1^, 2,957 cm^−1^, 2,930 cm^−1^, 2,868 cm^−1^, 1,694 cm^−1^; HRESIMS *m*/*z* 289.1448 [M-H]^-^ (calcd. for C_17_H_21_O_4_, 289.1434); for ^1^H-NMR (CD_3_OD, 600 MHz) and ^13^C-NMR (CD_3_OD, 100 MHz) data, see Table 1.

**TABLE 1:** ^1^H-NMR (600 MHz) and^13^C-NMR (100 MHz) data for compounds 1 and 2 (in CD_3_OD).

Position	Compound 1		Compound 2	
	*δ*_H_	*δ*_C_	*δ*_H_	*δ*_C_
1		198.7		198.8
2	2.60 (1H, dd, *J* = 16.3, 8.0 Hz, 2α)	50.1	2.61 (1H, dd, *J* = 16.2, 7.8 Hz, 2α)	50.1
	2.82 (1H, dd, *J* = 16.3, 3.8 Hz, 2β)		2.86 (1H, dd, *J* = 16.2, 3.5 Hz, 2β)	
3	4.24 (1H, m)	66.5	4.25 (1H, m)	66.5
4	2.92 (1H, dd, *J* = 15.9, 7.5 Hz, 4α)	40.5	2.92 (1H, dd, *J* = 16.1, 7.6 Hz, 4α)	40.5
	3.16 (1H, dd, *J* = 15.9, 3.8 Hz, 4β)		3.16 (1H, dd, *J* = 16.1, 3.6 Hz, 4β)	
4a		147.3		147.4
5	6.63 (1H, s)	114.6	6.63 (1H, s)	114.7
6		158.5		158.8
7		132.0		132.0
8		145.5		145.2
8a		124.0		124.0
9		207.7		207.7
10	2.47 (3H, s)	32.6	2.48 (3H, s)	32.6
11	2.89 (1H, m)	30.5	2.89 (2H, m)	30.0
	2.83 (1H, m)			
12	1.35 (2H, m)	41.3	1.51 (1H, m)	35.8
			1.32 (1H, m)	
13	1.62 (1H, m)	30.0	1.66 (1H, m)	37.5
14	0.92 (3H, d, *J* = 6.7 Hz)	22.6	3.35 (1H, m)	68.1
			3.48 (1H, m)	
15	0.92 (3H, d, *J* = 6.7 Hz)	22.6	0.96 (3H, d, *J* = 6.7 Hz)	16.8

#### Streptonaphthalene B (2)

White amorphous solid; [α] ${\;}^{20}{\;}_{\text{D}}$ −34 (*c* 0.1, MeOH); UV (MeOH) λ_max_ (log) 226 (4.33) nm, 278 (4.17) nm; CD (MeOH) 226 (Δ*ε* −3.76), 286 (Δ*ε* −1.33) nm; IR 3393 cm^−1^, 2,955 cm^−1^, 2,926 cm^−1^, 2,874 cm^−1^, 1,697 cm^−1^; HRESIMS *m*/*z* 305.1396 [M-H]^-^ (calcd. for C_17_H_21_O_5_, 305.1383); for ^1^H-NMR (CD_3_OD, 600 MHz) and ^13^C-NMR (CD_3_OD, 100 MHz) data, see Table 1.

#### Pestaloficin F (3)

Colorless oil; [α] ${\;}^{20}{\;}_{\text{D}}$ 73 (*c* 0.1, MeOH); UV (MeOH) λ_max_ (log) 202 (4.26) nm, 231 (3.61) nm; CD (MeOH) 202 (Δ*ε* −5.63), 231 (Δ*ε* 1.81) nm; IR 3287 cm^−1^, 2,955 cm^−1^, 2,868 cm^−1^, 1749 cm^−1^; HRESIMS *m*/*z* 213.1127 [M-H]^-^ (calcd. for C_11_H_17_O_4_, 213.1121); for ^1^H-NMR (CD_3_OD, 600 MHz) and ^13^C-NMR (CD_3_OD, 100 MHz) data, see Table 2.

**TABLE 2:** ^1^H-NMR (600 MHz) and ^13^C-NMR (100 MHz) data for compounds 3–5 (in CD_3_OD).

Position	Compound 3		Compound 4		Position	Compound 5	
	*δ*_H_	*δ*_C_	*δ*_H_	*δ*_C_		*δ*_H_	*δ*_C_
2		172.8		172.8	1	3.20 (1H, t, *J* = 7.1 Hz)	78.7
3		131.5		131.6	2	1.75 (2H, m)	35.0
4		160.2		160.2	3	3.67 (1H, m)	72.1
5	5.84 (1H, s)	99.7	5.84 (1H, s)	99.2	4	2.39 (1H, m)	34.0
1′	4.47 (1H, t, *J* = 6.7 Hz)	67.2	4.52 (1H, t, *J* = 6.3 Hz)	66.9	5	1.12 (1H, dd, *J* = 10.6, 4.1 Hz)	51.0
2′	1.76 (2H, m)	34.3	1.74 (2H, m)	37.0	6	4.01 (1H, t, *J* = 10.6 Hz)	70.1
3′	1.32 (1H, m)	35.5	1.39 (1H, m)	21.3	7	1.46 (1H, m)	55.4
	1.17 (1H, m)		1.48 (1H, m)				
4′	1.56 (1H, m)	28.9	1.49 (2H, m)	44.3	8	1.62 (1H, dq, *J* = 13.3, 3.5 Hz)	23.4
						1.17 (1H, m)	
5′	0.91 (3H, d, *J* = 6.6 Hz)	22.8		71.2	9	1.83 (1H, dt, *J* = 12.8, 3.5 Hz)	40.7
						1.04 (1H, dt, *J* = 12.8, 3.5 Hz)	
6′	0.91 (3H, d, *J* = 6.6 Hz)	22.7	1.17 (3H, s)	29.0	10		40.5
7′	2.10 (3H, s)	11.5	1.17 (3H, s)	28.9	11		75.5
8′			2.11 (3H, s)	11.5	12	1.21 (3H, s)	29.8
					13	1.27 (3H, s)	24.1
					14	0.91 (3H, d, *J* = 7.4 Hz)	8.5
					15	0.88 (3H, s)	15.6

#### Pestaloficin G (4)

Colorless oil; [α] ${\;}^{20}{\;}_{\text{D}}$ 117 (*c* 0.1, MeOH); UV (MeOH) λ_max_ (log) 206 (4.06) nm, 229 (3.54) nm; CD (MeOH) 206 (Δ*ε* −8.60), 229 (Δ*ε* 5.33) nm; IR 3372 cm^−1^, 2,928 cm^−1^, 2,860 cm^−1^, 1,599 cm^−1^; HRESIMS *m*/*z* 243.1236 [M-H]^-^ (calcd. for C_12_H_19_O_5_, 243.1227); for ^1^H-NMR (CD_3_OD, 600 MHz) and ^13^C-NMR (CD_3_OD, 100 MHz) data, see Table 2.

#### Eudesmanetetraiol A (5)

Yellow crystal; [α] ${\;}^{20}{\;}_{\text{D}}$ 96 (*c* 0.1, MeOH); IR 3443 cm^−1^; HRESIMS *m*/*z* 295.1873 [M + Na]^+^ (calcd. for C_15_H_28_O_4_Na, 295.1880); for ^1^H-NMR (CD_3_OD, 600 MHz) and ^13^C-NMR (CD_3_OD, 100 MHz) data, see Table 2.

#### Target Network Analysis

The ingredients isolated were imported into the PubChem database and ChemBio3D Ultra 14.0, and the 3D molecular structures were exported in the form of SDFs. The targets were retrieved from the online target prediction platform PharmMapper (http://www.lilab-ecust.cn/pharmmapper/). Human species was used for target prediction, and the targets with Norm Fit ≥ 0.75 were collected. Thereafter, the targets were converted to gene names using the UniProt Knowledgebase (UniProtKB, http://www.uniprot.org/), and species were restricted to “*Homo sapiens*.” Meanwhile, the NSCLC-related targets were obtained from the DisGeNET database (http://www.disgenet.org/) and TTD (http://database.idrb.cqu.edu.cn/TTD/). The STRING database (version 11.0, https://string-db.org/) was used to explore the protein–protein interactions (PPIs), and protein interactions with a confidence score > 0.4 were selected in the designed setting after eliminating duplicates and independent ones. Cytoscape software (version 3.7.2) was applied to construct the chemical–target network and protein–protein interaction (PPI) network. All genes were subjected to pathway enrichment analysis (KEGG analysis) using DAVID Bioinformatics Resources 6.8, and those pathway terms with a *p*-value < 0.05 were regarded as significant and interesting (Zhang et al., 2021).

## Results and Discussions

### Cytotoxic Effects of the Extract of AD16

**FIGURE 1:**
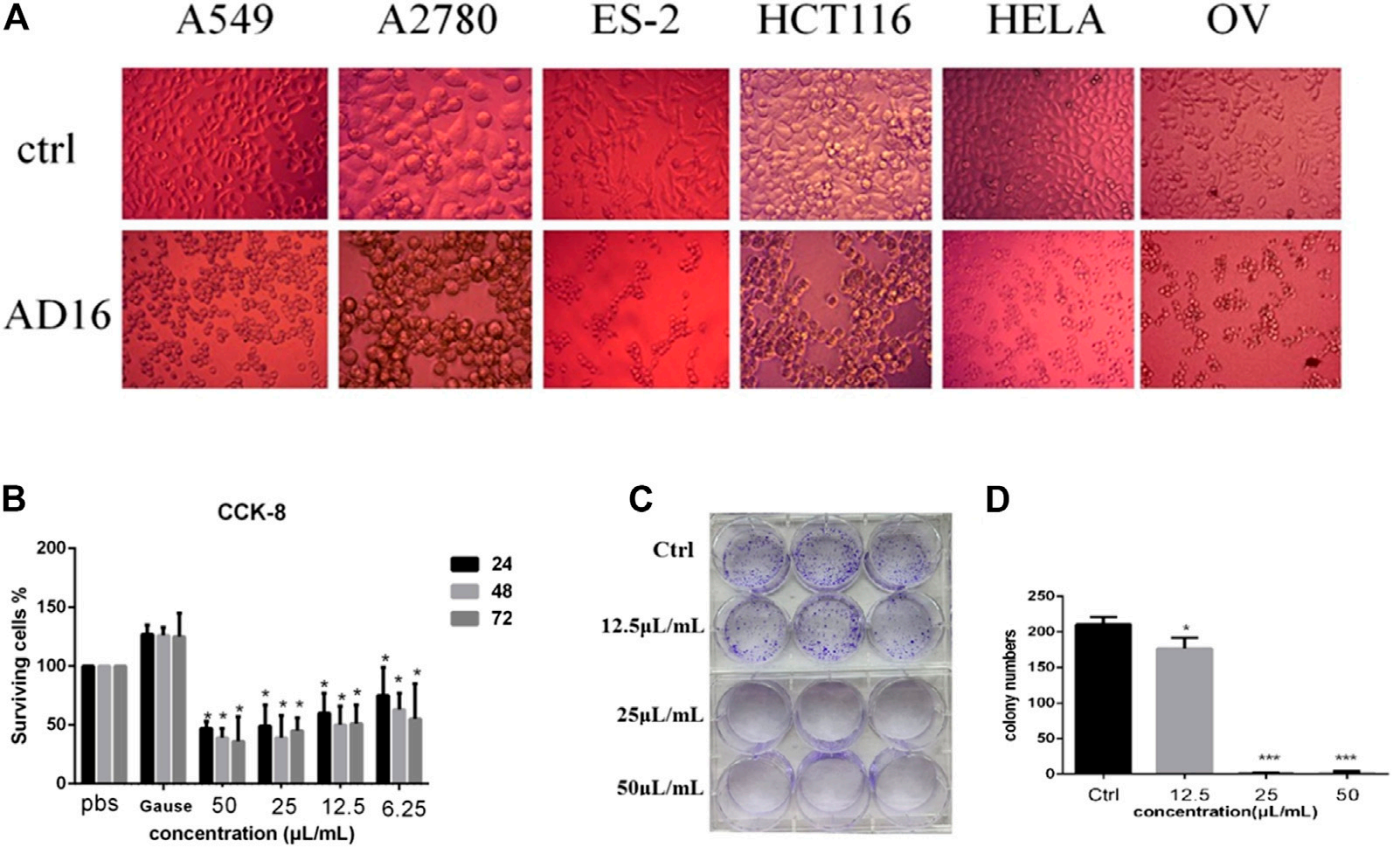
**(A)** Significant changes in cell morphology were observed under the microscope after AD16 metabolites were added in various cancer cells (×400). **(B)** CCK-8 assay results of AD16 metabolites in A549 cells. **(C)** Results of colony formation of A549 cells incubated with AD16 metabolites for 12 h. **(D)** The paired-sample t-test was used to analyze whether there was a significant difference in the number of colony formation between each AD16-added group and control (*p < 0.05, ***p < 0.001). Ctrl, control.

**FIGURE 2:**
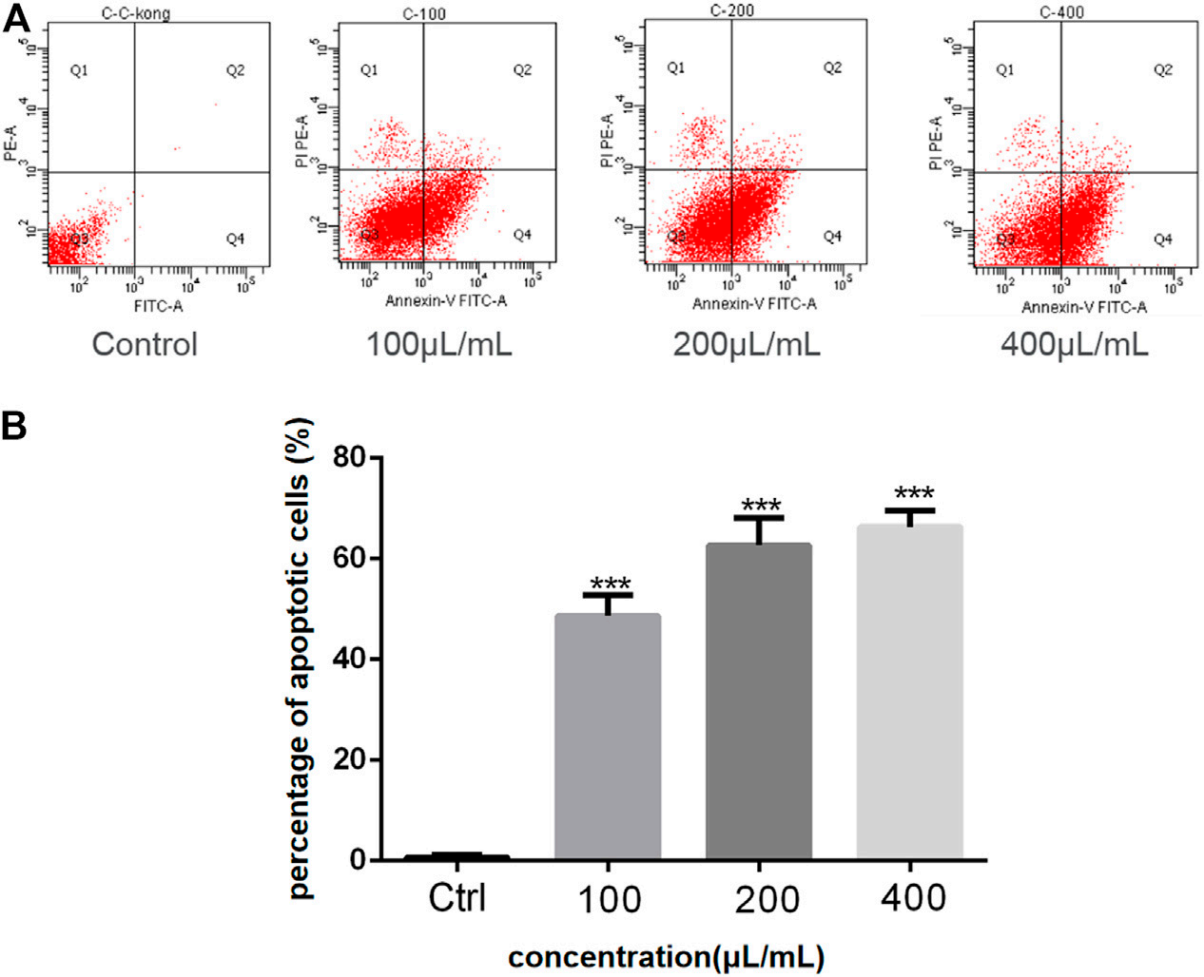
**(A)** The percent of apoptosis in A549 cells was evaluated by flow cytometry; **(B)** Student’s t-test was used to analyze the differences between the control group and the AD16 group (***p < 0.001). Ctrl, control.

The anticancer activity of the EtOAc extract of AD16 was investigated. Strain AD16 exhibited a broad killing spectrum of cancers including lung cancer (A549), ovarian cancer (A2780, ES-2/OV-90), colorectal cancer (HCT116), and cervical cancer (HeLa) at the concentration of 5 μL/ml (Figure 1A). The CCK-8 result of A549 cells incubated with AD16 demonstrated that the effects of AD16 were dose- and time-dependent against A549 as judged by cell proliferation percentages in comparison with the control (Figure 1B). The colony formation activity against A549 cells was also investigated, which indicated that AD16 could strongly inhibit colony formation of the A549 cell line (Figures 1C,D). To determine the possible mechanism of the anticancer effects of AD16, we detected the induction to apoptosis after treatment with AD16. Six hours after treatment with different concentrations, cells were double-stained with Annexin V and PI and subjected to flow cytometry to quantitatively analyze the apoptotic effects. As illustrated in Figure 2A, the percentages of total apoptotic cells, including the early apoptotic portion (Annexin V positive) and the late apoptotic portion (Annexin V and PI positive), were dose-dependently increased with increasing concentrations of AD16 in the A549 cell line (Figure 2B). These results suggested that the AD16 culture could suppress cell proliferation by inducing cell apoptosis.

### Cytotoxic Effect of the Subfractions

**FIGURE 3:**
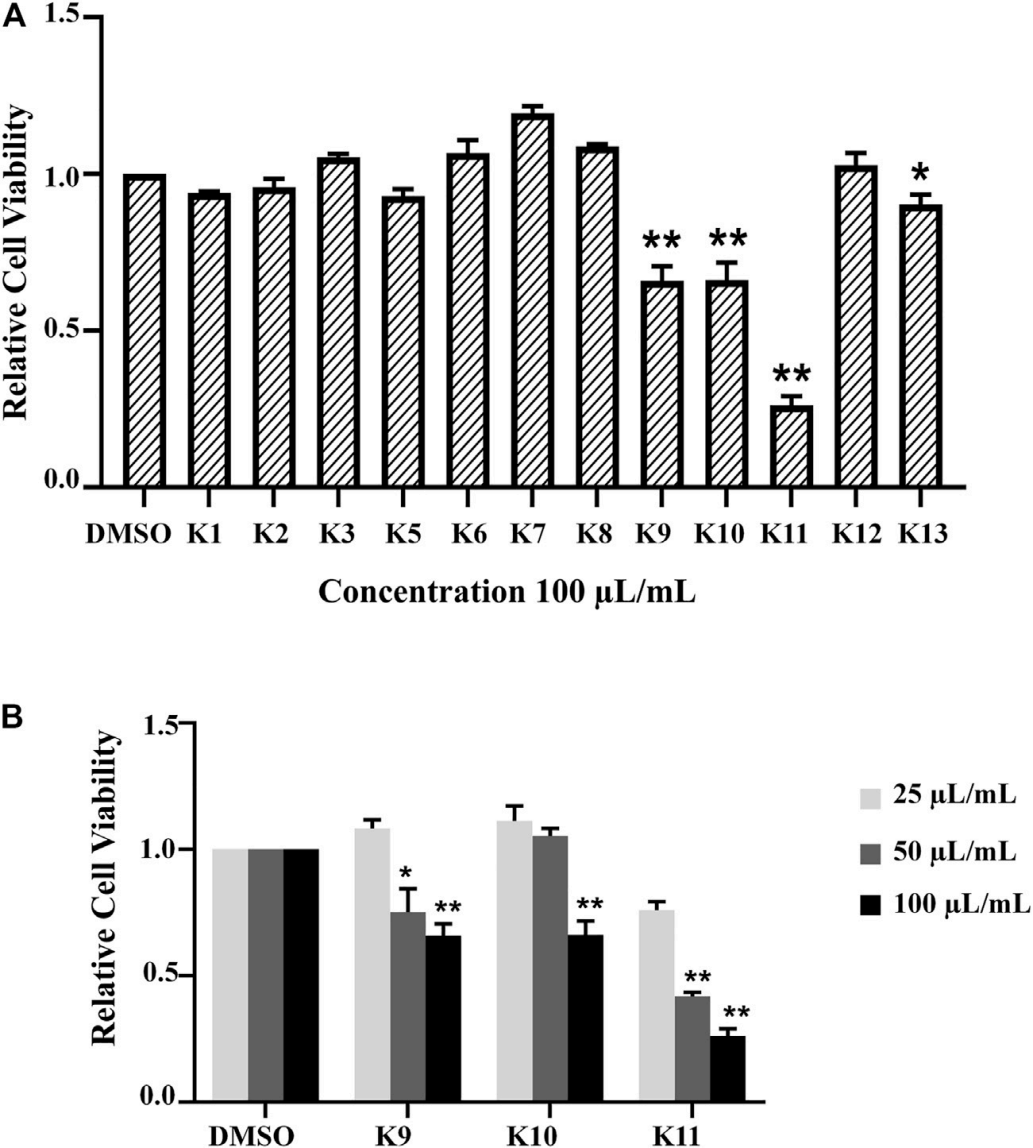
**(A)** All fractions showed different effects in the A549 cell line according to the CCK-8 assay 24 h after treatment with 100 μL/ml of K1–K13. **(B)** The activity was determined by the OD_492_ value compared with the control group. K9, K10, and K11 inhibited cancer cell activity in a concentration-dependent manner significantly (*p < 0.05, **p < 0.01).

Based on bioactivity-guided isolation, a large quantity of the AD16 extract was partitioned with ODS by MeOH–H_2_O gradients. All the fractions were examined to determine their anticancer effects at 100 μl/ml (Figures 3A,B). When compared to other fractions, fractions 9–11 showed the highest inhibitory activities (Figure 3B). Eventually, we isolated and identified 14 compounds, including five new compounds and nine previously known ones.

### Structural Determination of Compounds From AD16

**FIGURE 4:**
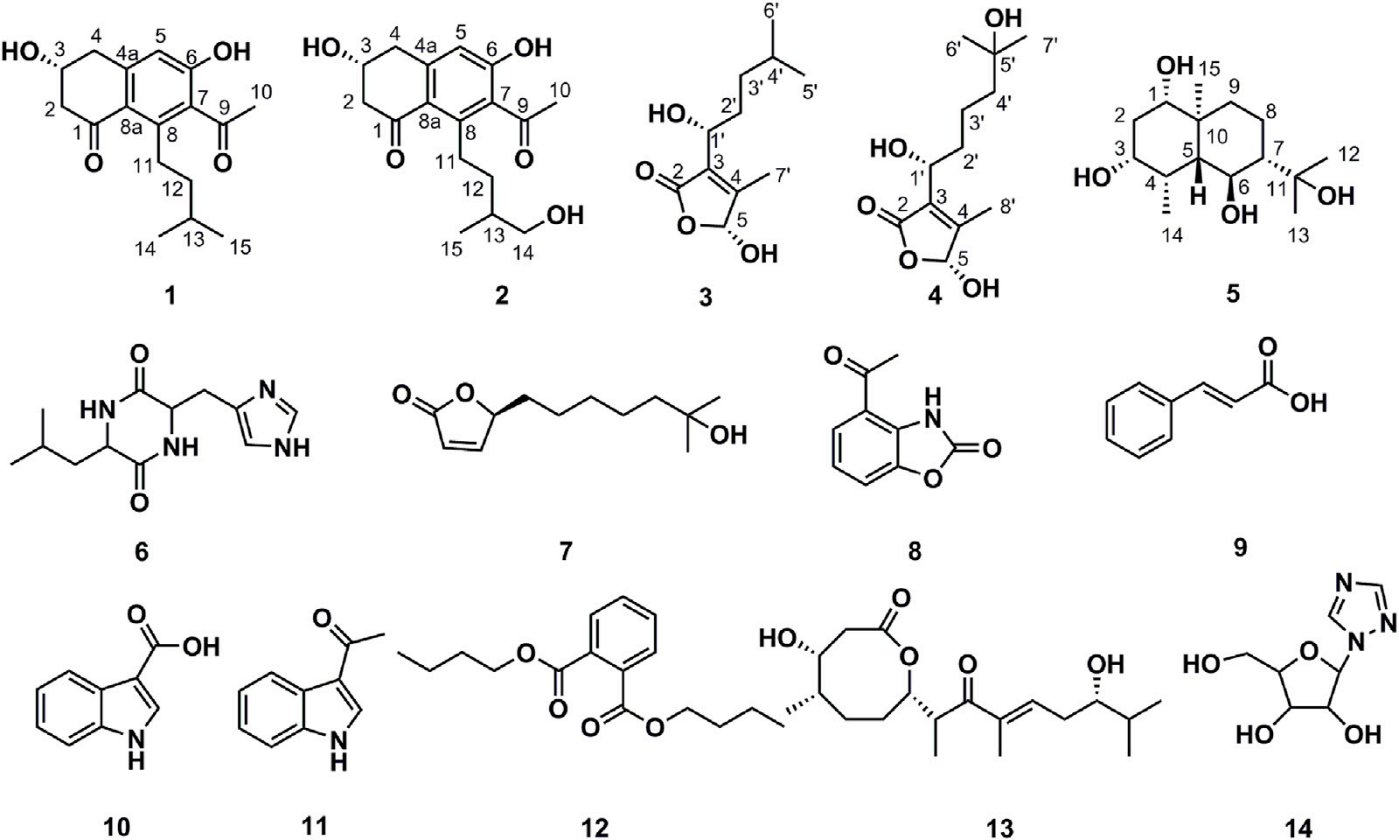
Chemical structures of compounds isolated from AD16.

**FIGURE 5:**
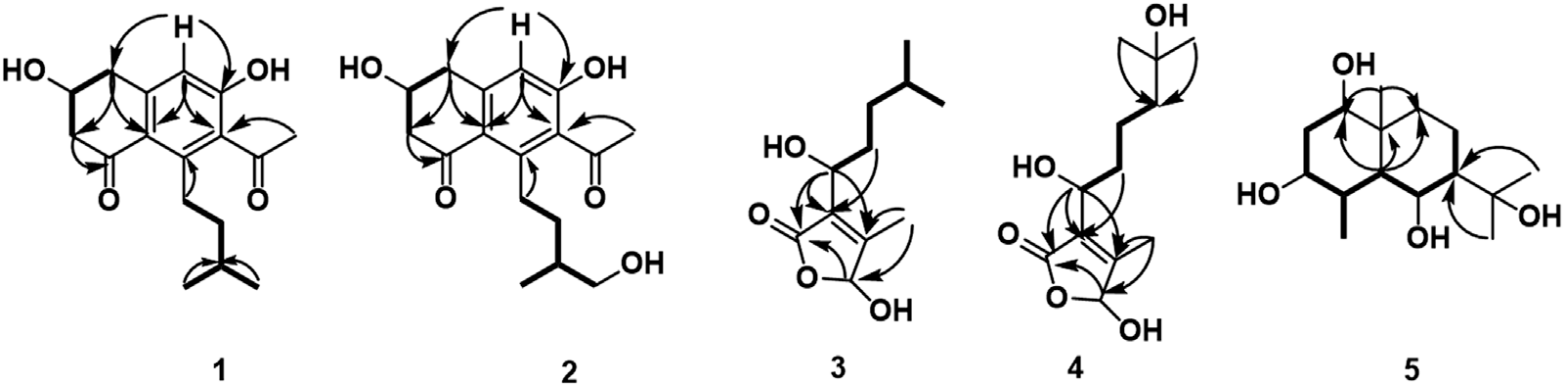
Key ^1^H–^1^H COSY (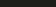) and HMBC (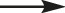) correlations of compounds 1–5.

**FIGURE 6:**
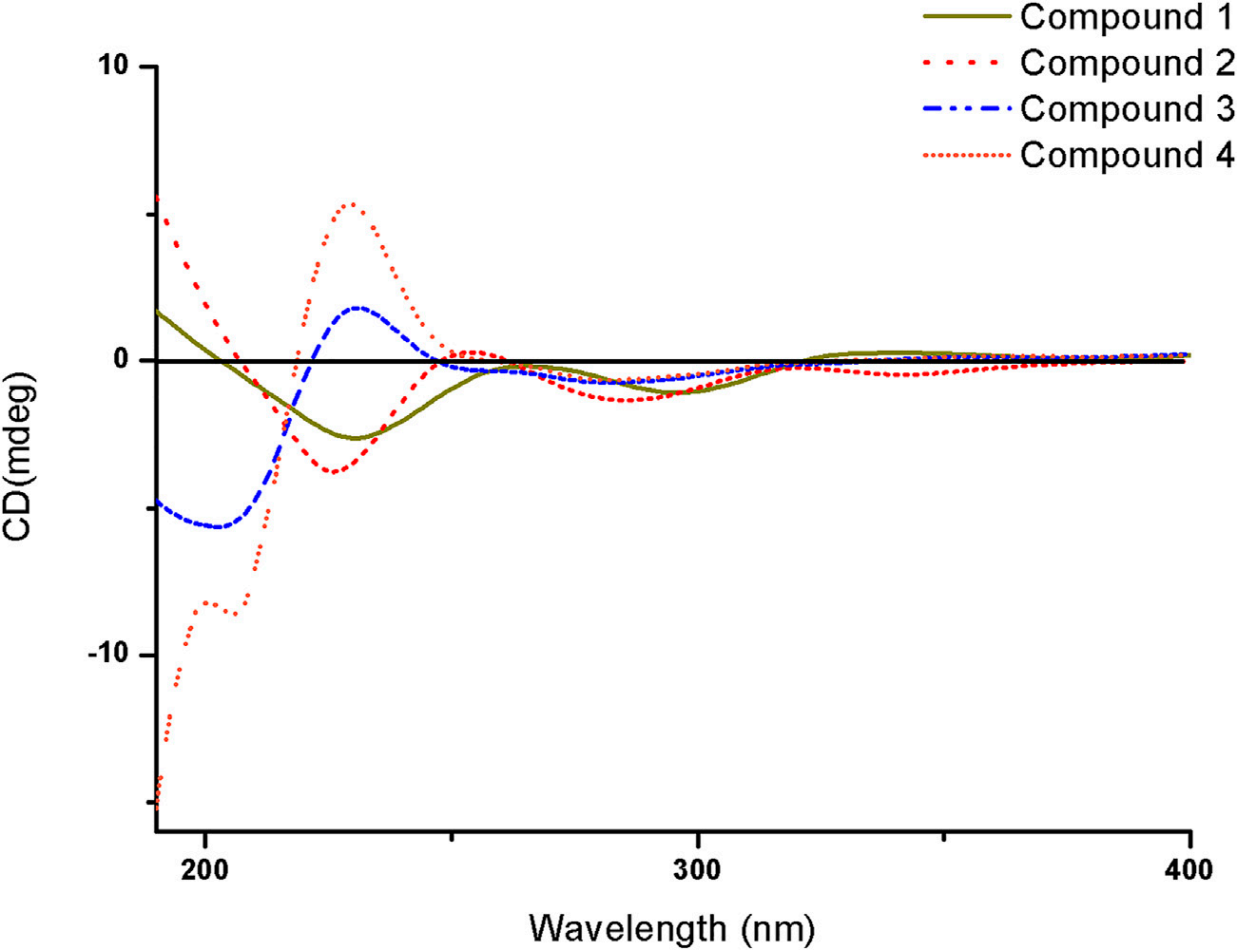
CD spectra of compounds 1–4.

Compound 1 was isolated as a white amorphous solid. The negative-ion ESIMS spectrum showed a peak at *m*/*z* 289.1448 [M-H]^-^, so its molecular formula was unambiguously assigned as C_17_H_22_O_4_ on the basis of HRESIMS data (Supplementary Figure S1-3). The ^1^H NMR spectrum of compound 1 showed one aromatic proton at *δ*_H_ 6.63 (1H, s, H-5), an oxygenated methine proton at *δ*_H_ 4.24 (1H, m, H-3), and three methyl groups at *δ*_H_ 2.47 (3H, s, H-10) and *δ*_H_ 0.92 (6H, d, *J* = 6.7 Hz, H-14/15). ^13^C NMR spectroscopic data revealed the presence of 17 carbon atoms, including two ketone carbonyls at*δ*_C_ 198.7 (C-1), 207.7 (C-9) and six aromatic carbon atoms at *δ*_C_ 158.5 (C-6), 147.3 (C-4a), 145.5 (C-8), 132.0 (C-7), 124.0 (C-8a), and 114.6 (C-5). The ^1^H NMR and ^13^C NMR data of 1 were very similar to those of the known compound 7-acetyl-3,6-dihydroxy-8-propyl-3,4-dihydronaphthalen-1(2H)-one (Yeo et al., 1998) (Supplementary Figures S1-1,2), except that the propyl moiety was replaced by the isopentyl moiety in 1 (Figure 4). Moreover, the ^1^H–^1^H COSY correlations between *δ*_H_ 2.89, 2.83 (H-11) and *δ*_H_ 1.35(H-12), *δ*_H_ 1.35 (H-12) and *δ*_H_ 1.62 (H-13), *δ*_H_ 1.62 (H-13), and *δ*_H_ 0.92 (H-14/15), as well as the HMBCs between H-14/15 (*δ*_H_ 0.92) and C-13 (*δ*_C_ 30.0), suggested the isopentyl fragment in 1. The HMBCs between H-11 (*δ*_H_ 2.89, 2.83) and C-8 (*δ*_C_ 145.5) suggested the isopentyl fragment to be located at C-8 in 1 (Figure 5) (Supplementary Figures S1-4,5,6). The configuration of the chiral carbon C-3 was assigned as *R* by comparing the CD spectrum (Figure 6) (negative Cotton effects at 230 and 296 nm) with that of 7-acetyl-3,6-dihydroxy-8-propyl-3,4-dihydronaphthalen-1(2H)-one (Huasin et al., 2012). Thus, compound 1 was named streptonaphthalene A.

Compound 2 was obtained as a white amorphous solid. Its molecular formula was determined as C_17_H_22_O_5_ on the basis of HRESIMS data, which gave a peak at *m*/*z* 305.1396 [M-H]^-^ (Supplementary Figure S2-3). The ^1^H NMR spectrum of compound 2 also showed one aromatic proton at *δ*_H_ 6.63 (1H, s, H-5), an oxygenated methine proton at *δ*_H_ 4.25 (1H, m, H-3), and two methyl groups at *δ*_H_ 2.48 (3H, s, H-10) and *δ*_H_ 0.96 (3H, d, *J* = 6.7 Hz, H-15) (Supplementary Figure S2-1). ^13^C NMR spectroscopic data revealed the presence of 17 carbon atoms. Analysis of ^1^H NMR and ^13^C NMR data indicated that compound 2 was similar to 1, except that the methyl group was replaced by the hydroxymethyl moiety (Supplementary Figures S2-2,4,5,6). The configuration of the carbon C-3 was deduced to be *R* by comparing the structure with that of 1, which might be derived from the same biosynthesis pathway. Unfortunately, because of its limited amount, the configuration of C-13 was not further determined by the chemical method. Compound 2 was named streptonaphthalene B.

Compound 3 was obtained as colorless oil. Its molecular formula was deduced as C_11_H_18_O_4_, from the HRESIMS signal at *m*/*z* 213.1127 [M-H]^-^ (calcd. for C_11_H_17_O_4_, 213.1121) (Supplementary Figure S3-3). The ^1^H NMR spectrum of compound 3 showed two oxygenated methine protons at *δ*_H_ 5.84 (1H, s, H-5) and *δ*_H_ 4.47 (1H, t, *J* = 6.7 Hz, H-1′) (Supplementary Figure S3-1). The ^13^C NMR spectrum revealed the presence of 11 carbon atoms (Supplementary Figure S3-2). Its NMR spectra contained resonances reminiscent of a 5-hydroxy-2(5H)-furanone skeleton carrying an alkane moiety. The furanone moiety was determined by the chemical shifts of the two quaternary carbon atoms *δ*_C_ 131.5 (C-3), *δ*_C_ 160.2 (C-4), the carboxy carbon (*δ*_C_ 172.8, C-2), and the methylene carbon (*δ*_C_ 99.7, C-5), as well as the HMBCs of *δ*_H_ 5.84 (H-5) with *δ*_C_ 172.8 (C-2). ^1^H–^1^H COSY correlations of *δ*_H_ 4.47 (H-1′)/*δ*_H_ 1.76 (H-2′), *δ*_H_ 1.76 (H-2′)/*δ*_H_ 1.32, 1.17 (H-3′), *δ*_H_ 1.32, 1.17 (H-3′)/*δ*_H_ 1.56 (H-4′), and *δ*_H_ 1.56 (H-4′)/*δ*_H_ 0.91 (H-5′/6′) enabled the deduction of the C-6 alkane moiety. The HMBCs of *δ*_H_ 4.47 (H-1′) with *δ*_C_ 131.5 (C-3)/172.8 (C-2)/160.2 (C-4), *δ*_H_ 1.76 (H-2′) with *δ*_C_ 131.5 (C-3), and *δ*_H_ 2.10 (H-7′) with *δ*_C_ 160.2 (C-4)/131.5 (C-3)/99.7 (C-5) confirmed the location of the alkane moiety at C-3 and the methyl moiety at C-4 (Supplementary Figures S3-4,5,6). In the NOESY spectrum, the correlations of *δ*_H_ 2.10 (7′-CH_3_) with *δ*_H_ 4.47 (H-1′) and *δ*_H_ 5.84 (H-5) indicated that the two protons H-1′ and H-5 were in the same orientation (Supplementary Figure S3-7). The negative Cotton effect at 202 nm (Figure 6) was in good agreement with those of the model compound with 5*R* configuration, indicating the 5*R*, 1′*R* configuration of 3 (Song et al., 2018). Thus, the structure of 3 was assigned as shown in Figure 4, named pestaloficin F.

Compound 4 was obtained as colorless oil. Its molecular formula was deduced as C_12_H_20_O_5_ by analysis of its HRESIMS data (*m*/*z* 243.1236 [M-H]^-^, calcd. for C_12_H_19_O_5_, 243.1227) (Supplementary Figure S4-3). The comparison of the NMR spectroscopic data of 4 with those of 3 indicated that 4 also had one butenolide moiety (Supplementary Figures S4-1,2). The ^1^H–^1^H COSY correlations of *δ*_H_ 4.52 (H-1′)/*δ*_H_ 1.74 (H-2′), *δ*_H_ 1.74 (H-2′)/*δ*_H_ 1.39 (H-3′), *δ*_H_ 1.39 (H-3′)/*δ*_H_ 1.49 (H-4′) and HMBCs of *δ*_H_ 1.17 (H-6′/H-7′)/*δ*_C_ 71.2 (H-5′), *δ*_H_ 1.49 (H-4′)/*δ*_C_ 71.2 (H-5′) enabled the deduction of the alkyl moiety (Figure 5) (Supplementary Figure S4-4). The HMBCs of *δ*_H_ 4.52 (H-1′) with *δ*_C_ 131.6 (C-3)/172.8 (C-2), *δ*_H_ 1.74 (H-2′) with *δ*_C_ 131.6 (C-3), and *δ*_H_ 2.11 (H-8′) with *δ*_C_ 160.2 (C-4)/131.6 (C-3)/99.2 (C-5) confirmed the location of the alkane moiety at C-3 and the methyl moiety at C-4 (Supplementary Figures S4-5,6,7). The CD spectrum of 4 showed similar CEs to 3 (Figure 6), indicating the 5*R*, 1′*R* configuration of 4. Compound 4 was named pestaloficin G.

Compound 5 was obtained as yellow gum. Its molecular formula was deduced as C_15_H_28_O_4_ by analysis of its HRESIMS data (*m*/*z* 295.1873 [M + Na]^+^, calcd. for C_15_H_28_O_4_Na, 295.1880) (Supplementary Figure S5-3). The ^1^H NMR spectrum of 5 showed signals of three three-proton singlets at *δ*_H_ 0.88 (3H, s), 1.21 (3H, s), and 1.27 (3H, s) for methyl groups attached to quaternary carbon atoms, one three-proton doublet at *δ*_H_ 0.91 (3H, d, *J* = 7.4 Hz) for the methyl group attached to methine carbon, three methylene protons at *δ*_H_ 1.04 (1H, dt, *J* = 12.8, 3.5 Hz) and 1.83 (1H, dt, *J* = 12.8, 3.5 Hz), 1.17 (1H, m) and 1.62 (1H, dq, *J* = 13.5, 3.5 Hz), and 1.75 (2H, m), three methine proton (bearing hydroxyl groups) signals at *δ*_H_ 3.20 (1H, t, *J* = 7.1 Hz), 3.67 (1H, m), and 4.01 (1H, t, *J* = 10.6 Hz), and three methine proton signals at *δ*_H_ 1.12 (1H, dd, *J* = 10.6, 4.1 Hz), 1.46 (1H, m), and 2.39 (1H, m) (Supplementary Figure S5-1). The ^13^C-NMR and DEPT spectra of 5 showed 15 carbon signals (Supplementary Figure S5-2). C-1 (*δ*_C_ 78.7) was connected to C-2 (*δ*_C_ 35.0) to C-8 (*δ*_C_ 23.4) based on the ^1^H–^1^H COSY correlations of H-1 (*δ*_H_ 3.20)/H-2 (*δ*_H_ 1.75)/H-3 (*δ*_H_ 3.67)/H-4 (*δ*_H_ 2.39)/H-5 (*δ*_H_ 1.12)/H-6 (*δ*_H_ 4.01)/H-7 (*δ*_H_ 1.46)/H-8 (*δ*_H_ 1.62), and HMBC of H-5 (*δ*_H_ 1.12) with C-1 (*δ*_C_ 78.7), C-9 (*δ*_C_ 40.7), and C-10 (*δ*_C_ 40.5), CH_3_-15 (*δ*_H_ 0.88) with C1 (*δ*_C_ 78.7) and C9 (*δ*_C_ 40.7), and CH_3_-12/13 (*δ*_H_ 1.21/1.27) with C7 (*δ*_C_ 55.4) enabled the deduction of the planner structure of compound 5 (Figure 5) (Supplementary Figures S5-4,5,6,7), which had an eudesmane skeleton of sesquiterpene (Katsutani et al., 2020).

The relative stereochemistry of 5 was deduced from the analysis of its NOESY correlations. H-6 (*δ*_H_ 4.01) showed strong NOE interactions with CH_3_-13 (*δ*_H_ 1.27), CH_3_-14 (*δ*_H_ 0.91), and CH_3_-15 (*δ*_H_ 0.88); at the same time, NOE correlations were observed in H-1 (*δ*_H_ 3.20) with H-3 (*δ*_H_ 3.67) and H-5 (*δ*_H_ 1.12), but H-1 and H-3 showed no correlations with H-14 and H-15, suggesting that H-6 (*δ*_H_ 4.01), CH_3_-13 (*δ*_H_ 1.27), CH_3_-14 (*δ*_H_ 0.91), and CH_3_-15 (*δ*_H_ 0.88) should be placed as α orientation and H-1 (*δ*_H_ 3.20), H-3 (*δ*_H_ 3.67), H-4 (*δ*_H_ 2.39), H-5 (*δ*_H_ 1.12), and H-7 (*δ*_H_ 1.46) should be placed as β orientation. Thus, the structure of 5 was established unambiguously. Compound 5 was named eudesmanetetraiol A.

### Network Pharmacology Analysis

**FIGURE 7:**
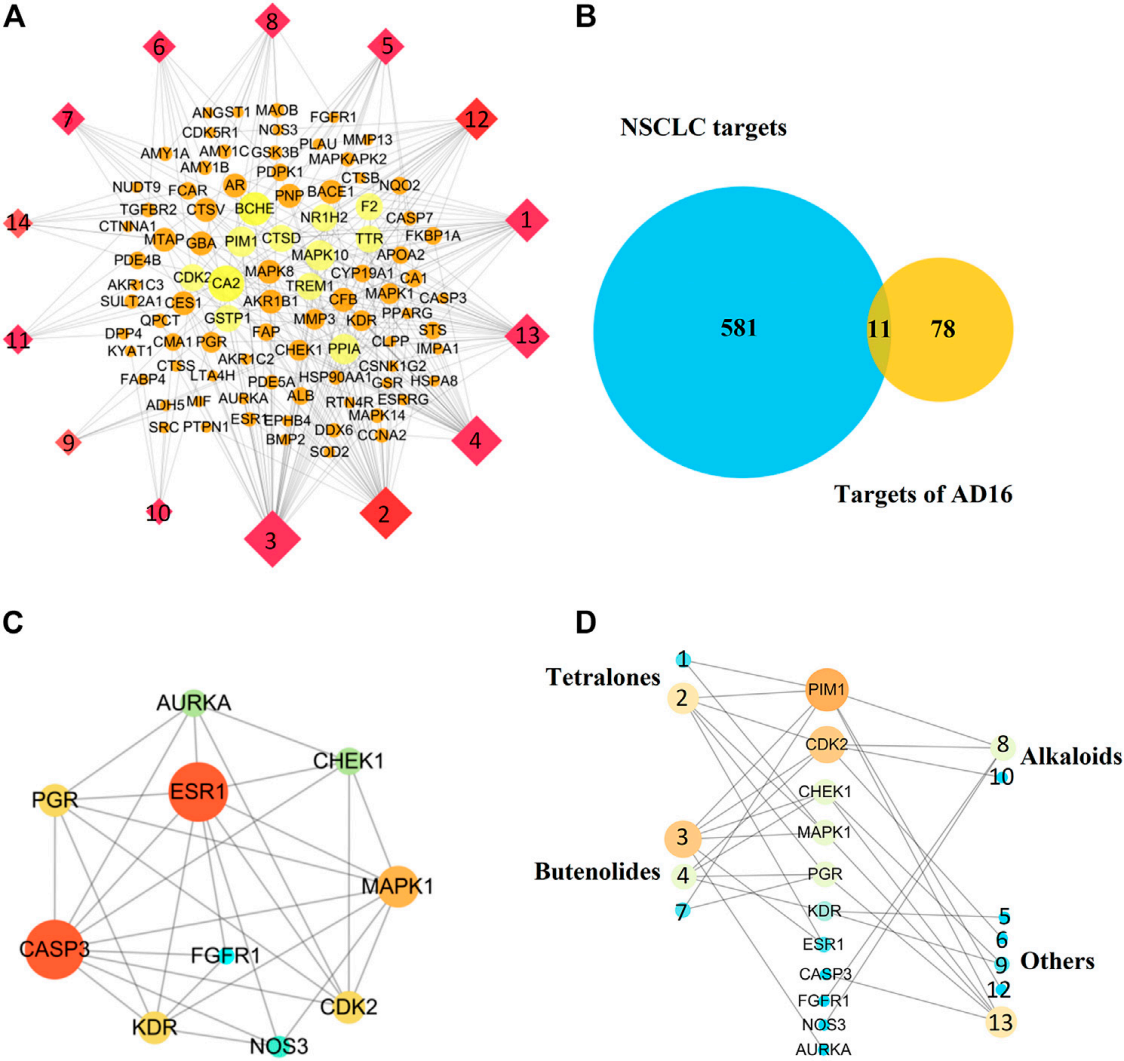
**(A)** Component–target network of AD16; **(B)** Venn diagram of targets between AD16-related targets and NSCLC targets; **(C)** protein–protein interactions between the targets of AD16; **(D)** interactions between components and NSCLC targets.

Network pharmacology is a systems biology–based methodology focused on the complex interaction network composed of diseases, genes, protein targets, and drugs using holistic and systemic views in a biological system, offering an effective strategy to uncover the overall action mode of multiple compounds (Bu et al., 2021; Tu et al., 2021). Therefore, to predict the underlying mechanism of AD16, a network pharmacology approach was applied. All the isolated compounds were used for target prediction, and the targets with the probability more than 0.75 were used for analysis. As a result, a total of 89 targets were summarized. The target–compound network was constructed as well (Figure 7A). The DisGeNET database and TTD search was performed to predict 592 targets associated with NSCLC. Then, eleven targets were screened out by looking for the overlapping targets from the compound-related targets and NSCLC-related targets (Figure 7B). The connections of the targets are shown in Figure 7C. Ten targets were identified in the PPI network based on their topological parameters. The gene products AURKA, CHEK1, PGR, ESR1, MAPK1, CASP3, FGFR1, CDK2, KDR, and NOS3 with high node degree were considered the key targets of AD16 against NSCLC. Among them, three targets with a higher degree value among the anti-NSCLC activity of AD16 are caspase 3 (CASP3), estrogen receptor 1 (ESR1), and mitogen-activated protein kinase 1 (MAPK1).

It can be seen from the results of the interactions between components of AD16 and NSCLC targets (Figure 7D) that 12 in 14 ingredients could correspond to multiple targets within multiple pathways, which were responsible for the anti-NSCLC effect of AD16. The compounds pestaloficin F (3), streptonaphthalene B (2), and 4-hydroxy-8-[6-hydroxy-1,3,7-trimethyl-2-oxo-oct-3-enyl]-5-methyl-oxocan-2-one (13) having the highest degree value (6, 5, 5), which attribute nodes in the network graph, could be considered the core ingredients in the network with a major anti-NSCLC effect. Over the years, butenolides and tetralones have played an important role in drug discovery, design, and development of plentiful pharmacologically active moieties. A lot of natural butenolides have been isolated from endophytic fungus and other microbial sources, which covered a broad range of therapeutic activities, including anticancer effects (Kornsakulkarn.et al., 2011; Kil et al., 2018; Husain et al., 2019; Wang et al., 2019; Yang et al., 2019). Compounds 3 (a new butenolide) and 2 (a new tetralone), proposed to be active constituents of AD16 herein, could act as leading compounds for further structural modification and drug design.

In addition, our result showed that cinnamic acid (9) played function on the target KDR. According to the references, 9 significantly increased the ratio of tumor growth inhibition, mean survival time, and percentage of the lifespan of the treated mice (Almeer et al., 2019). Furthermore, 9 induced angiogenesis *in vivo* and *in vitro*, which is related to VEGF and Flk-1/KDR expressions of endothelial cells (Choi et al., 2009). It was also reported that DBP (12) could inhibit the PI3K/Akt signaling pathway in INS-1 cells to induce cell apoptosis (Li et al., 2021). These results partially supported these biological processes predicted by network pharmacology.

**FIGURE 8:**
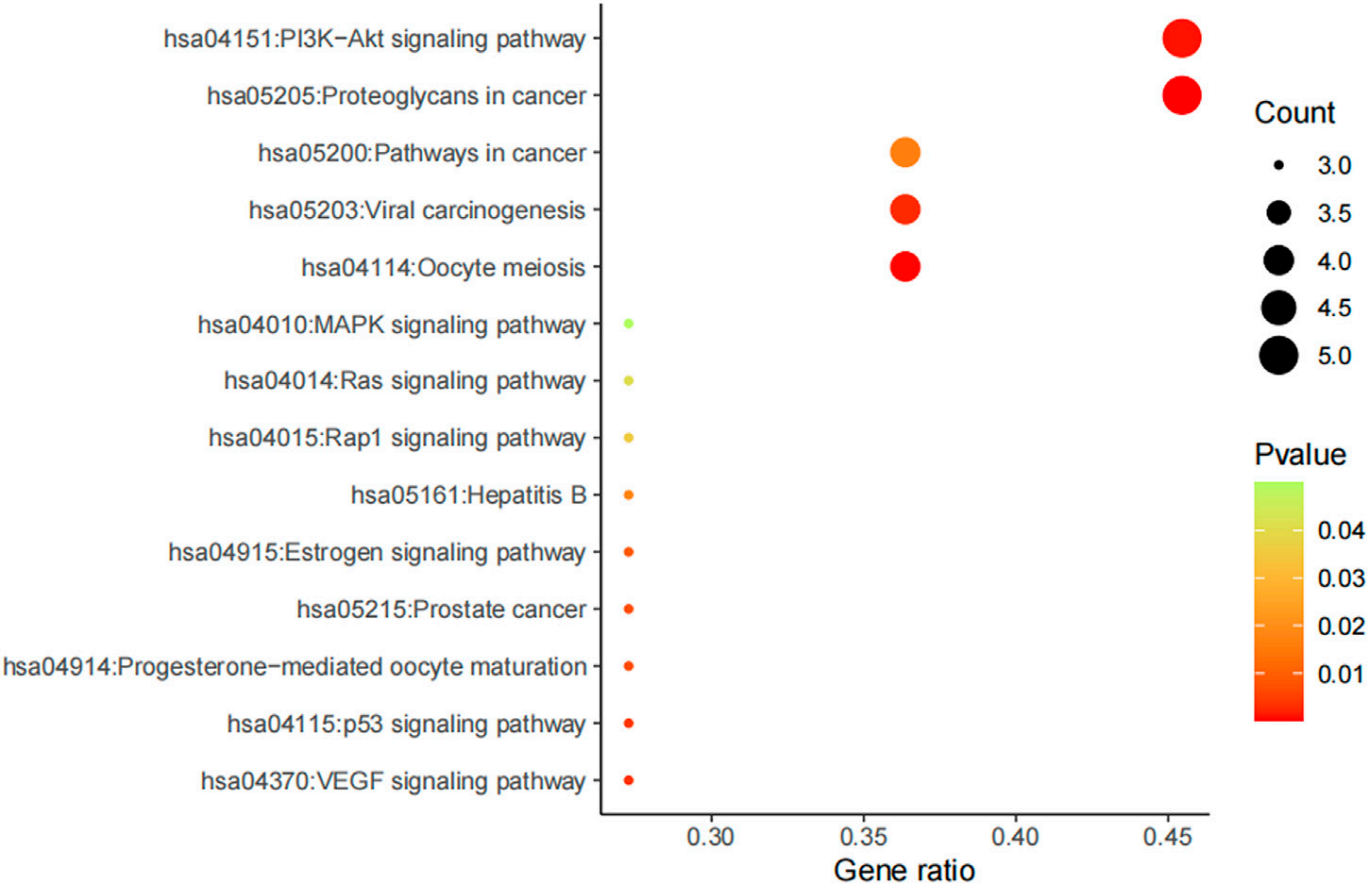
KEGG result of AD16 against NSCLC.

Furthermore, potential regulated biological processes and signaling pathways of AD16 treatment were predicted by KEGG analysis, and anti-NSCLC–related signal pathways were summarized (Figure 8). In addition to the PI3K–Akt signaling pathway and proteoglycans in cancers, pathways in cancer, viral carcinogenesis, and oocyte meiosis were the other main patterns for AD16 to achieve its anti-NSCLC effects (Zhang et al., 2017; Chen et al., 2018; Kim et al., 2018).

To summarize, we isolated one bacterial strain AD16 from human gut microbiota that had significant cytotoxic effects on A549. Fourteen compounds were isolated and identified by various chromatographic methods. Among them, five compounds were new, and their structures were determined by NMR, HRESIMS, and CD methods. However, as the amount of components isolated was limited, we inferred the anti-NSCLC mechanism of the AD16 compounds mainly based on network pharmacology. Network pharmacology analysis revealed that the regulation of AD16 on NSCLC could be via acting on multiple targets, multiple pathways, and multiple biological processes. Compounds 3, 2, and 13 might possibly be the key components of AD16 for its anti-NSCLC effects. In addition, the PI3K–Akt signaling pathway and proteoglycans in cancer pathway were the main patterns for AD16 to achieve its anti-NSCLC effects. Our work demonstrated the function mechanism of the human gut bacterial strain AD16 by secondary metabolites’ identification, network pharmacology, and experimental validation. It not only expanded the chemical and pharmacological diversities of metabolites from gut microbiota but also recommended that gut microbiota is of great potential for the discovery of new anticancer agents.

## Data Availability

We obtained a written informed consent from participant or their guardian, consistent with the 1975 Declaration of Helsinki. All experimental protocols were reviewed and approved by The Ethics Committee, Harbin Medical University, and all experiments were performed in accordance with relevant guidelines and regulations.
