# Supplementary material for: Dissection of the Functional Mechanism of Human Gut Bacterial Strain AD16 by Secondary Metabolites’ Identification, Network Pharmacology, and Experimental Validation
Source: Front Pharmacol. 2021 Nov 5;12:706220. doi: 10.3389/fphar.2021.706220 (PMC8602878; doi:10.3389/fphar.2021.706220)
Supplement: Supplementary file 1 [file DataSheet1.docx]

Supplementary Material

## Supplementary Figures

S1-1. ^1^H NMR spectrum of streptonaphthalen A (1).

S1-2. ^13^C NMR spectrum of streptonaphthalen A (1).

S1-3. HRESIMS spectrum of streptonaphthalen A (1).

S1-4. ^1^H, ^1^H-COSY spectrum of streptonaphthalen A (1).

S1-5. HSQC spectrum of streptonaphthalen A (1).

S1-6. HMBC spectrum of streptonaphthalen A (1).

S2-1. ^1^H NMR spectrum of streptonaphthalen B (2).

S2-2. ^13^C NMR spectrum of streptonaphthalen B (2).

S2-3. HRESIMS spectrum of streptonaphthalen B (2).

S2-4. ^1^H, ^1^H-COSY spectrum of streptonaphthalen B (2).

S2-5. HSQC spectrum of streptonaphthalen B (2).

S2-6. HMBC spectrum of streptonaphthalen B (2).

S3-1. ^1^H NMR spectrum of pestaloficin F (3).

S3-2. ^13^C NMR spectrum of pestaloficin F (3).

S3-3. HRESIMS spectrum of pestaloficin F (3).

S3-4. ^1^H, ^1^H-COSY spectrum of pestaloficin F (3).

S3-5. HSQC spectrum of pestaloficin F (3).

S3-6. HMBC spectrum of pestaloficin F (3).

S3-7. NOESY spectrum of pestaloficin F (3).

S4-1. ^1^H NMR spectrum of pestaloficin G (4).

S4-2. ^13^C NMR spectrum of pestaloficin G (4).

S4-3. HRESIMS spectrum of pestaloficin G (4).

S4-4. ^1^H, ^1^H-COSY spectrum of pestaloficin G (4).

S4-5. HSQC spectrum of pestaloficin G (4).

S4-6. HMBC spectrum of pestaloficin G (4).

S4-7. NOESY spectrum of pestaloficin G (4).

S5-1. ^1^H NMR spectrum of eudesmanetetraiol A (5).

S5-2. ^13^C NMR spectrum of eudesmanetetraiol A (5).

S5-3. HRESIMS spectrum of eudesmanetetraiol A (5).

S5-4. ^1^H, ^1^H-COSY spectrum of eudesmanetetraiol A (5).

S5-5. HSQC spectrum of eudesmanetetraiol A (5).

S5-6. HMBC spectrum of eudesmanetetraiol A (5).

S5-7. NOESY spectrum of eudesmanetetraiol A (5).


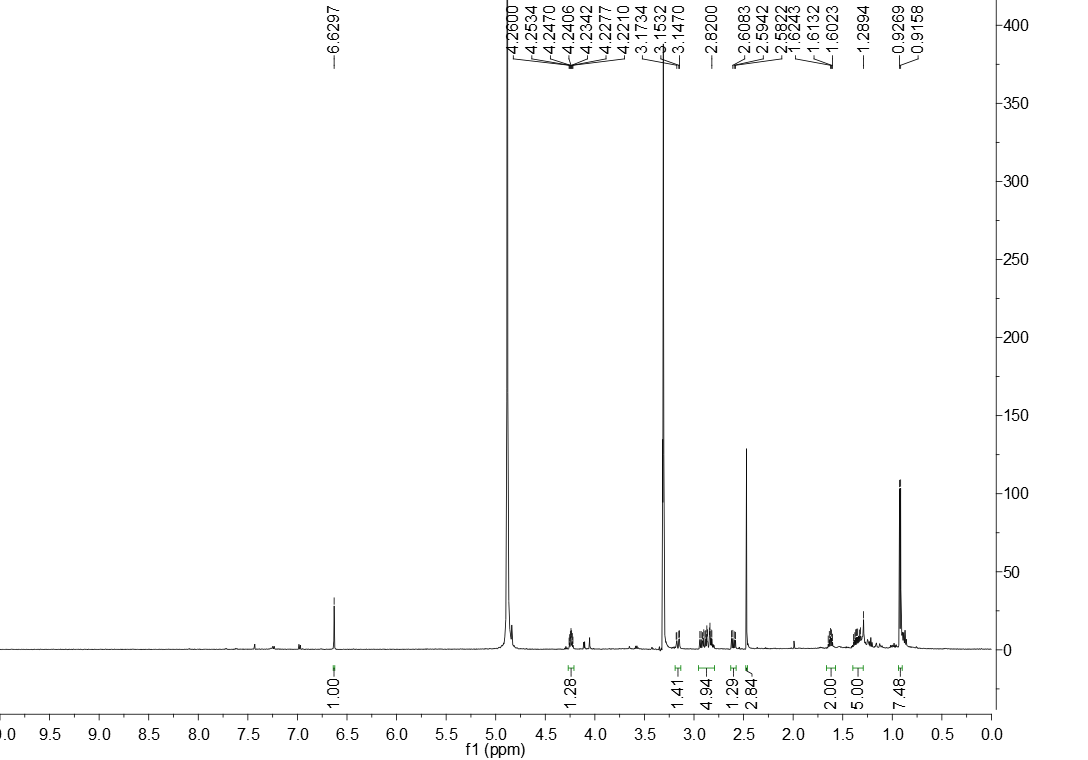
S1-1. ^1^H NMR spectrum of streptonaphthalen A (1).


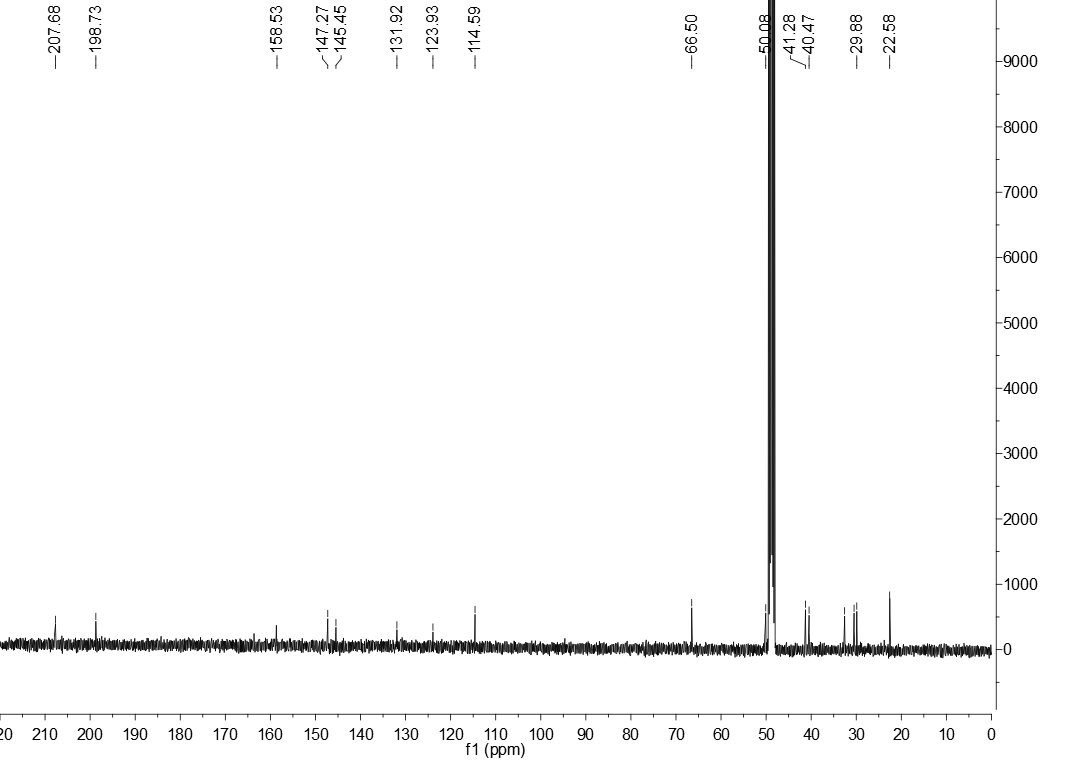


S1-2. ^13^C NMR spectrum of streptonaphthalen A (1).

S1-3. HRESIMS spectrum of streptonaphthalen A (1).


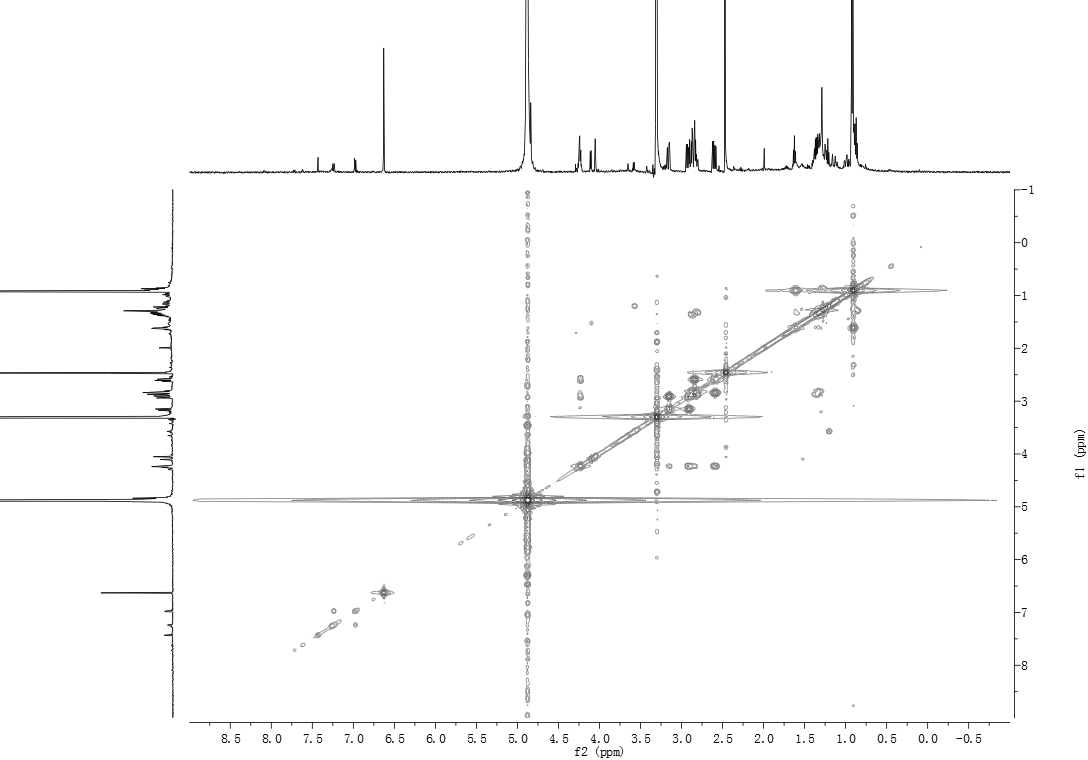


S1-4. ^1^H, ^1^H-COSY spectrum of streptonaphthalen A (1).


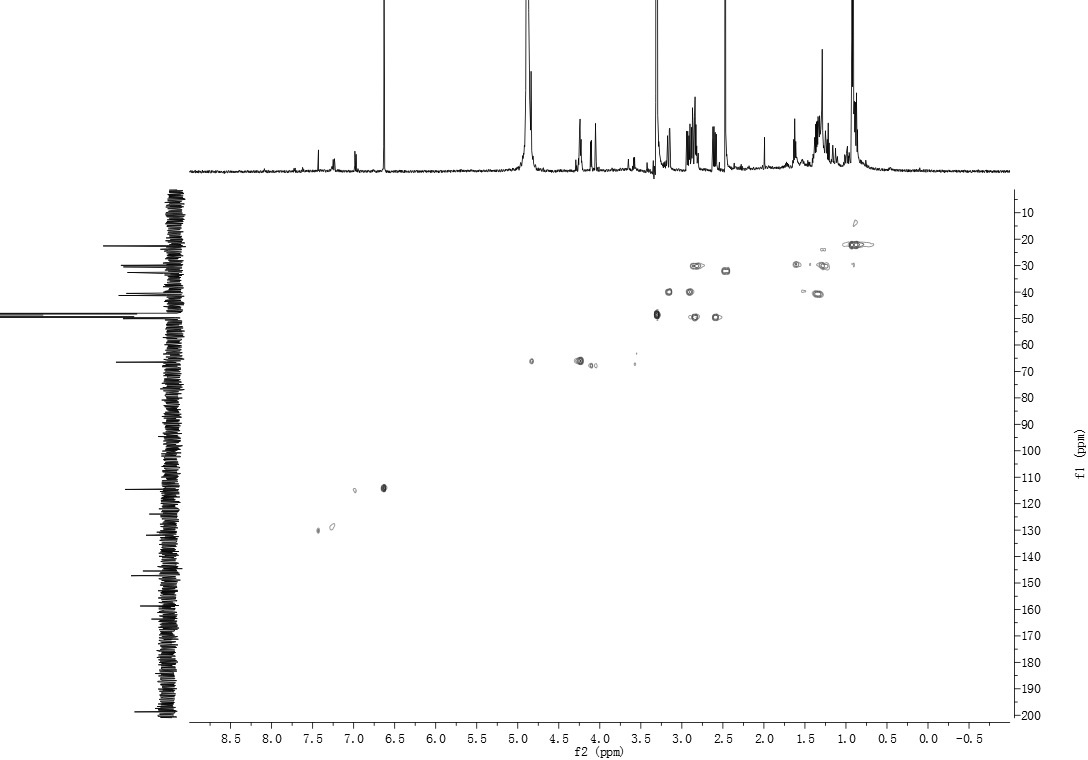
S1-5. HSQC spectrum of streptonaphthalen A (1).


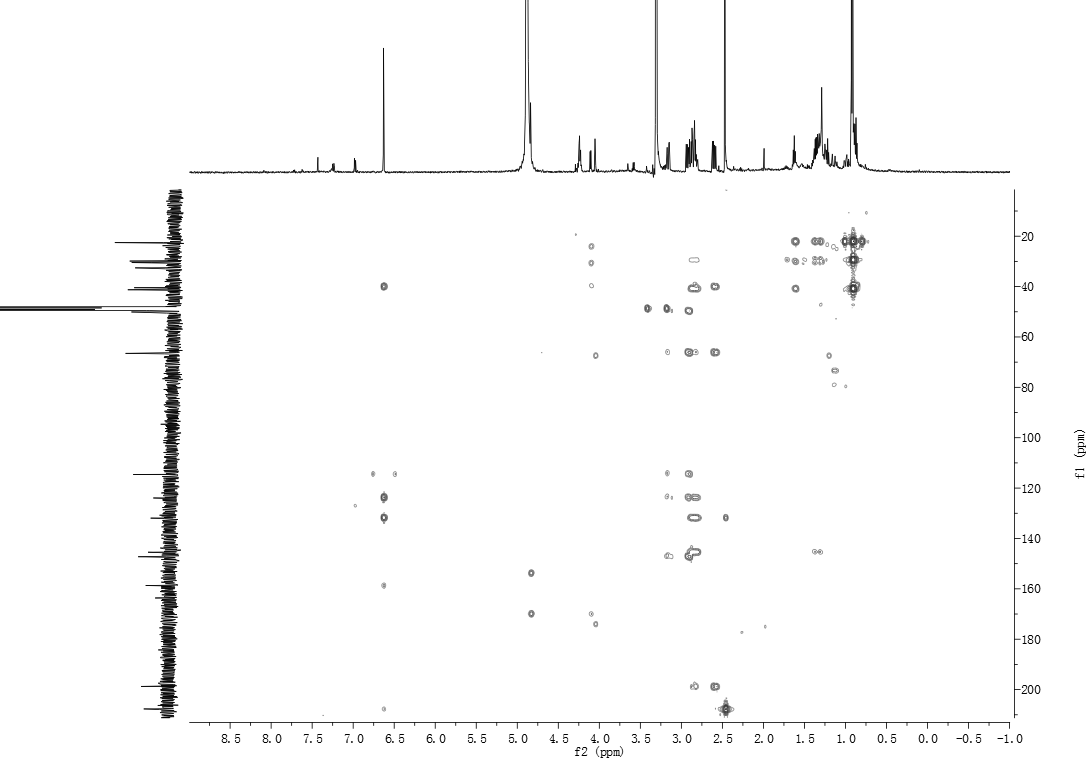


S1-6. HMBC spectrum of streptonaphthalen A (1).


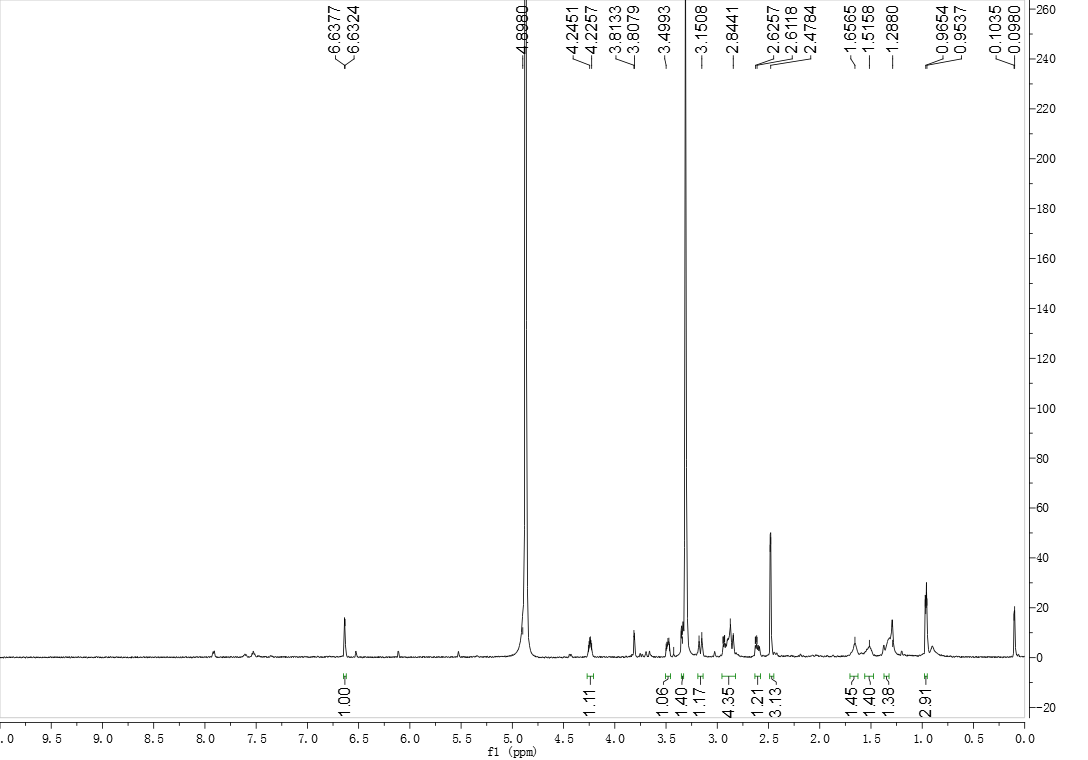


S2-1. ^1^H NMR spectrum of streptonaphthalen B (2).


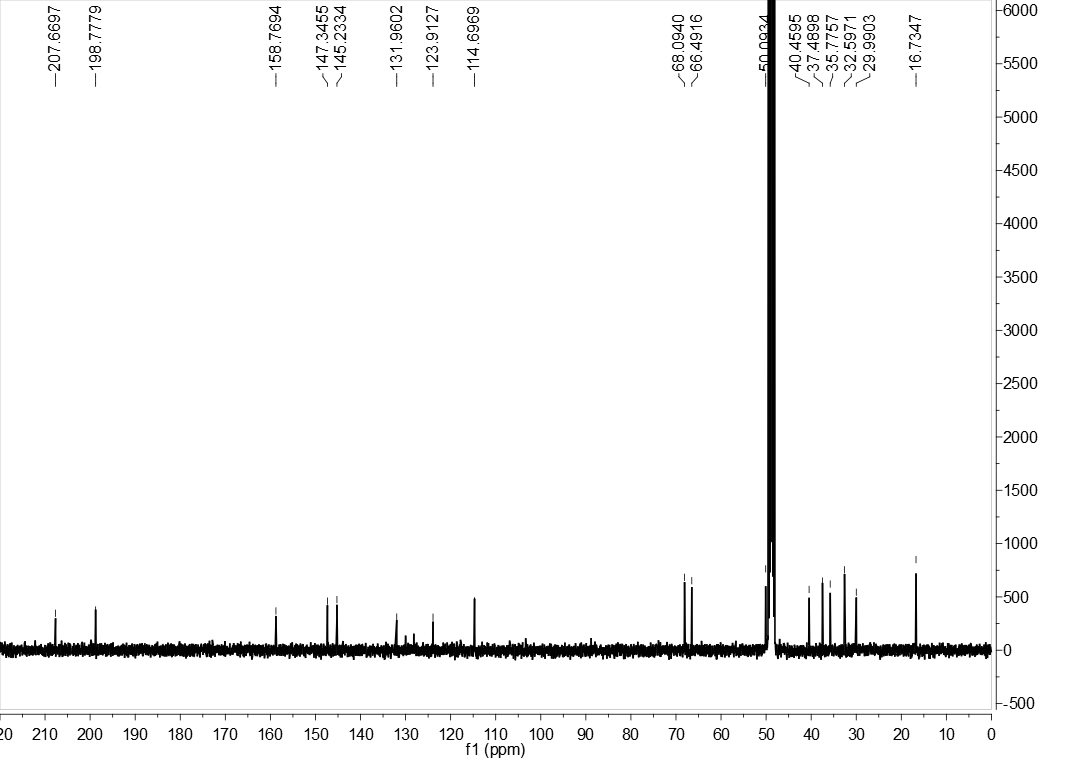


S2-2. ^13^C NMR spectrum of streptonaphthalen B (2).

S2-3. HRESIMS spectrum of streptonaphthalen B (2).


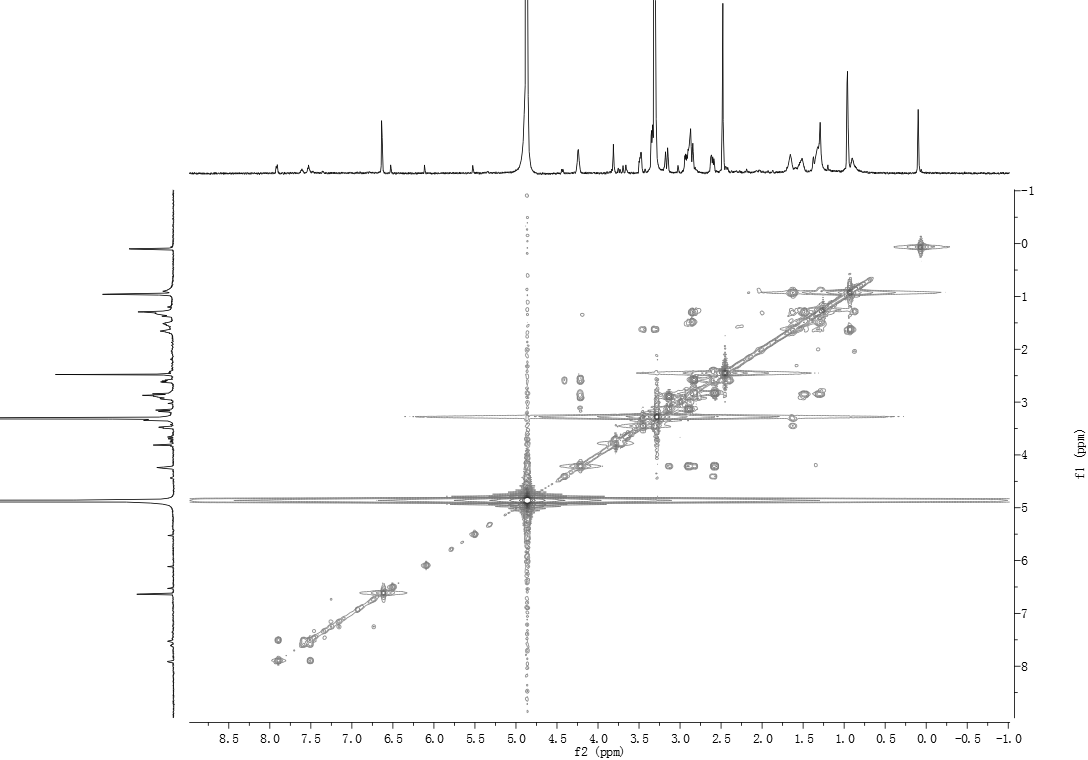


S2-4. ^1^H, ^1^H-COSY spectrum of streptonaphthalen B (2).


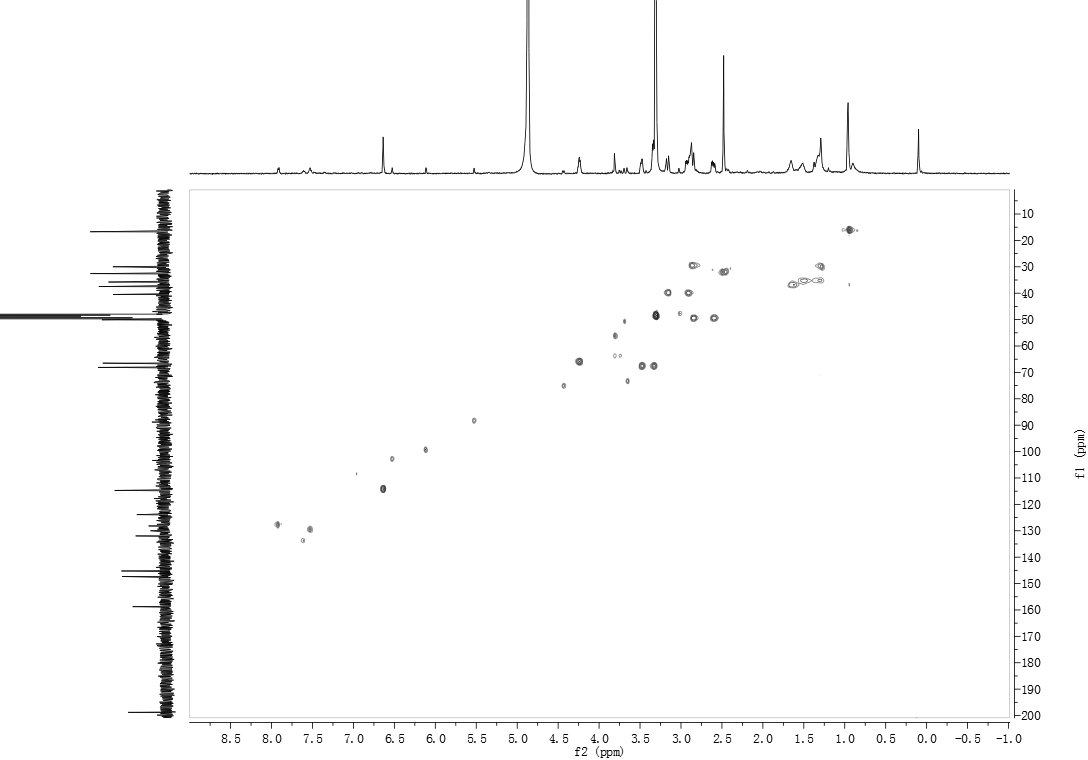
S2-5. HSQC spectrum of streptonaphthalen B (2).


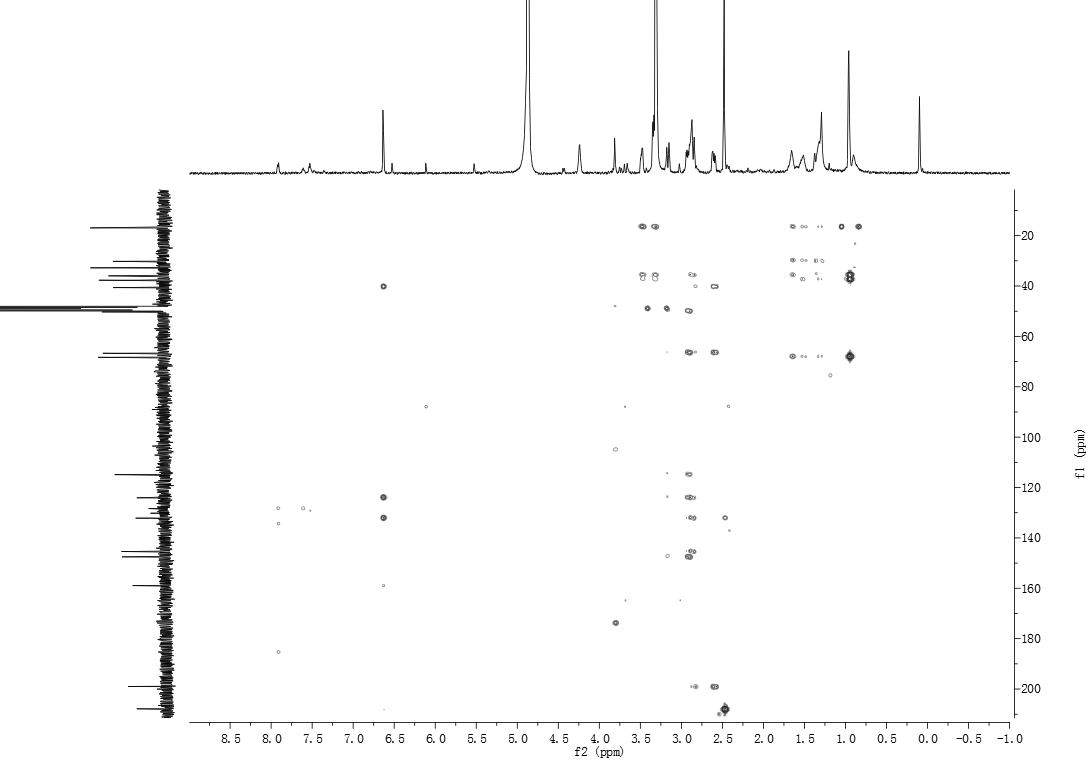


S2-6. HMBC spectrum of streptonaphthalen B (2).


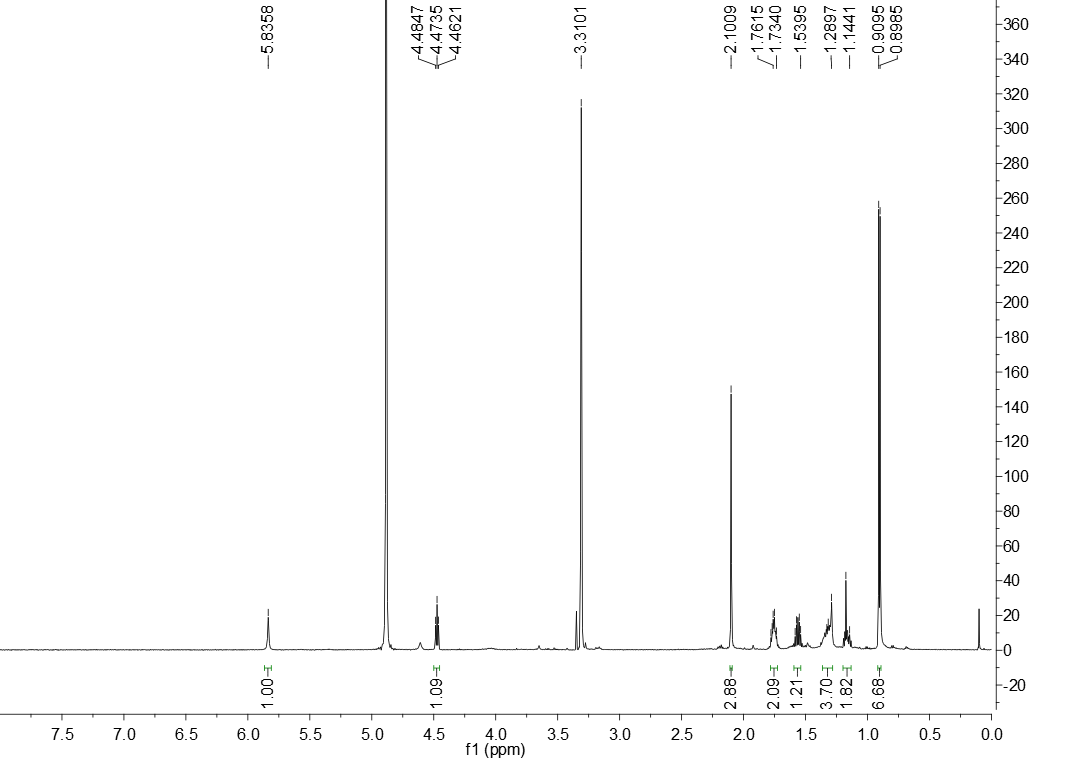
S3-1. ^1^H NMR spectrum of pestaloficin F (3).


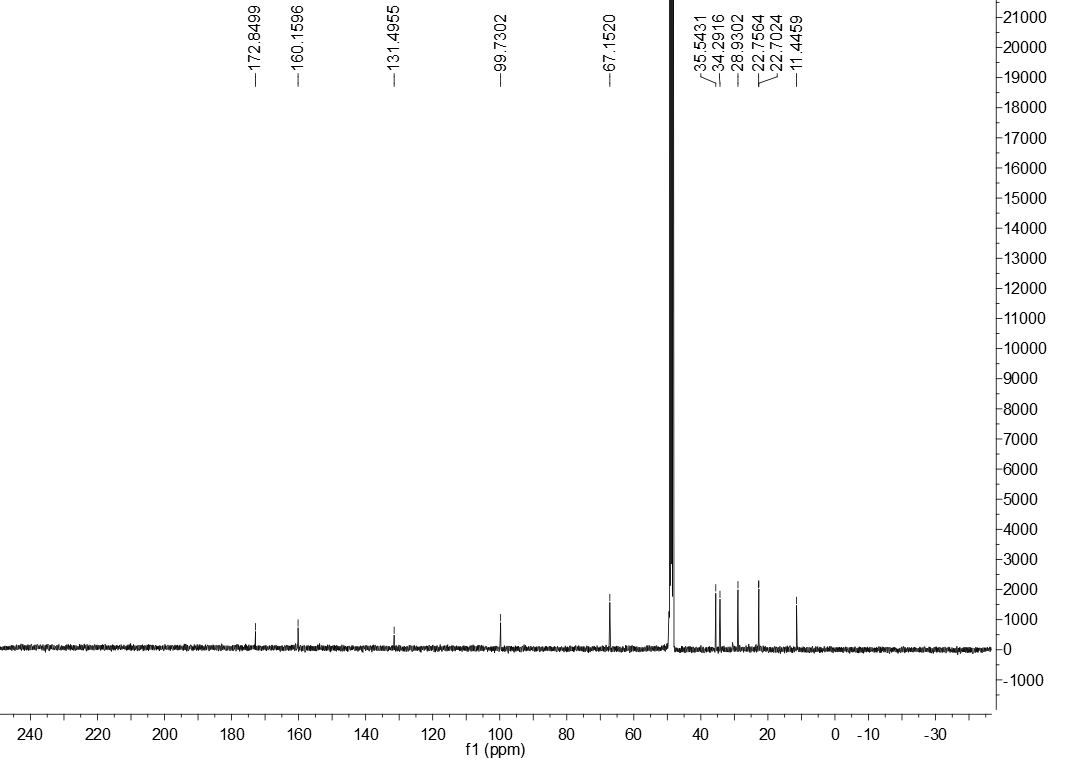


S3-2. ^13^C NMR spectrum of pestaloficin F (3).

S3-3. HRESIMS spectrum of pestaloficin F (3).


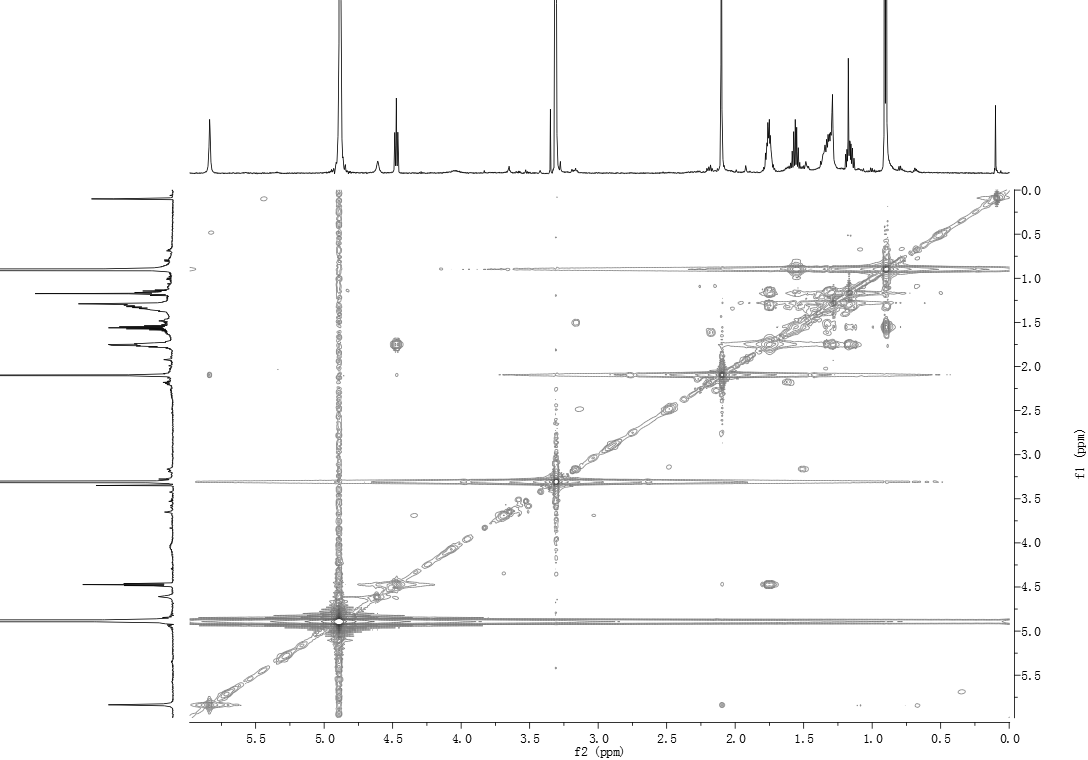


S3-4. ^1^H, ^1^H-COSY spectrum of pestaloficin F (3).


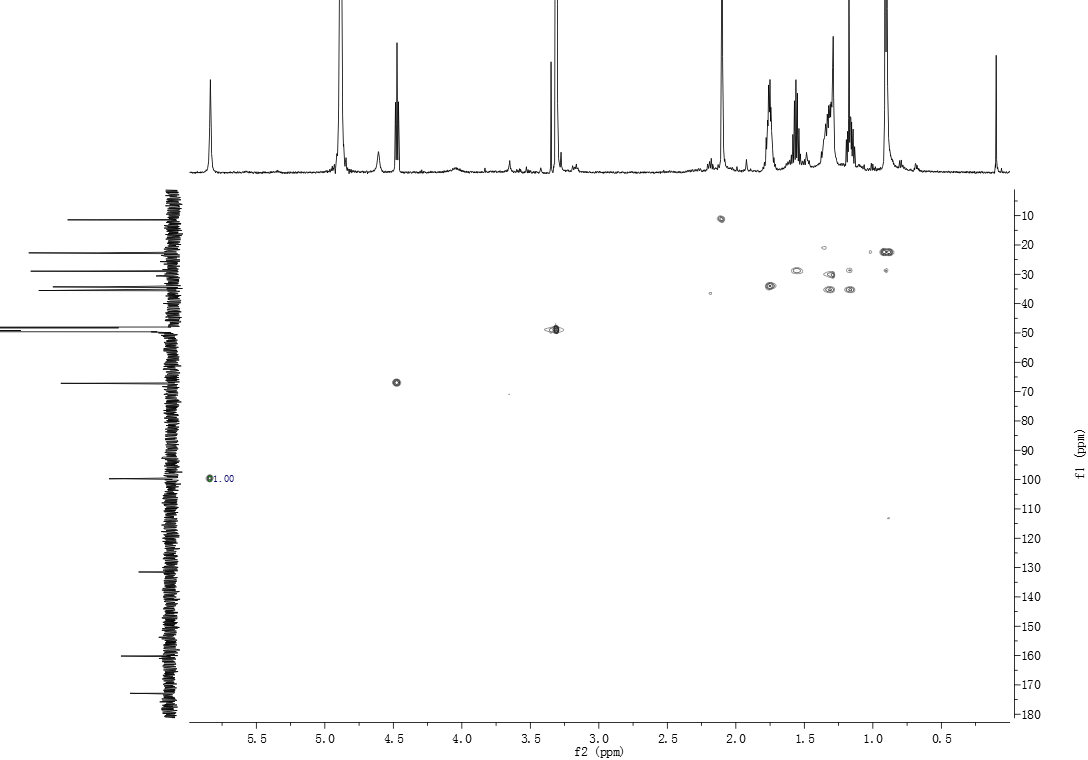


S3-5. HSQC spectrum of pestaloficin F (3).


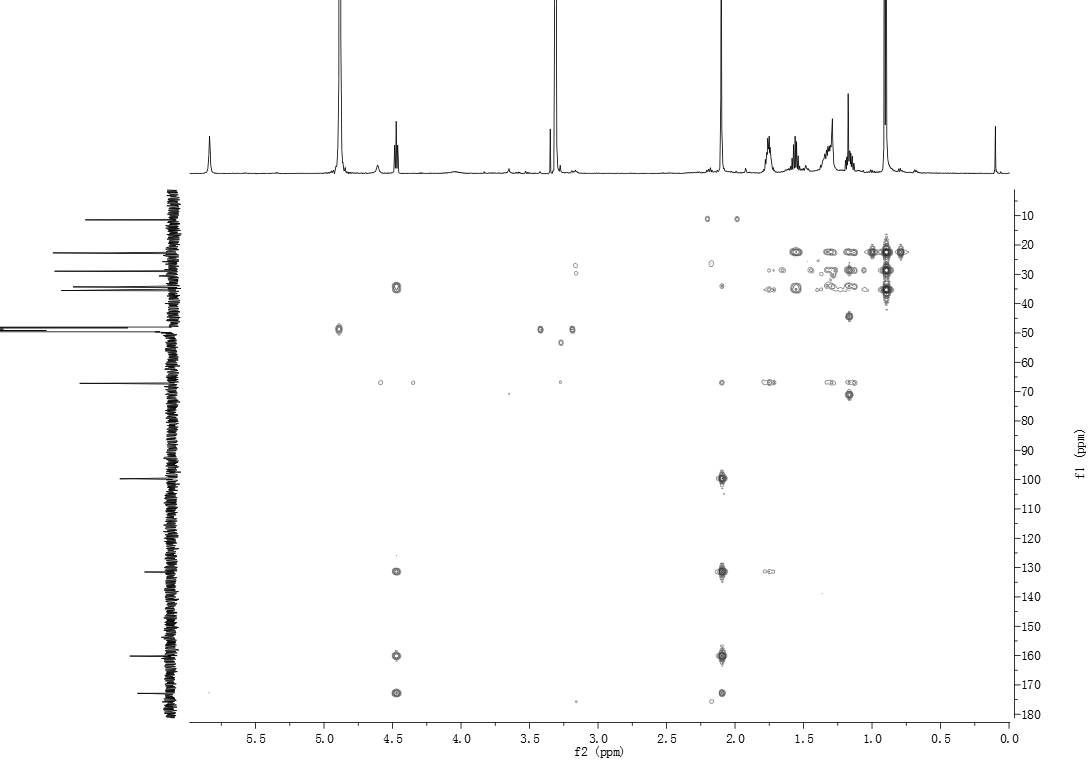


S3-6. HMBC spectrum of pestaloficin F (3).


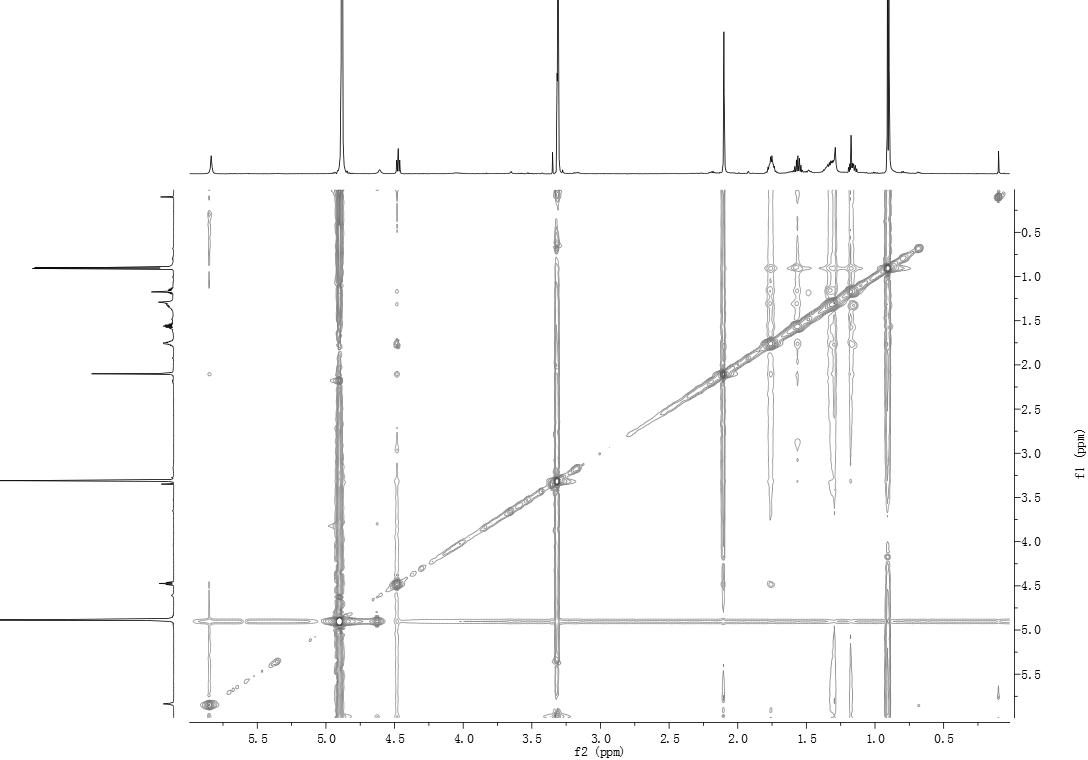


S3-7. NOESY spectrum of pestaloficin F (3).


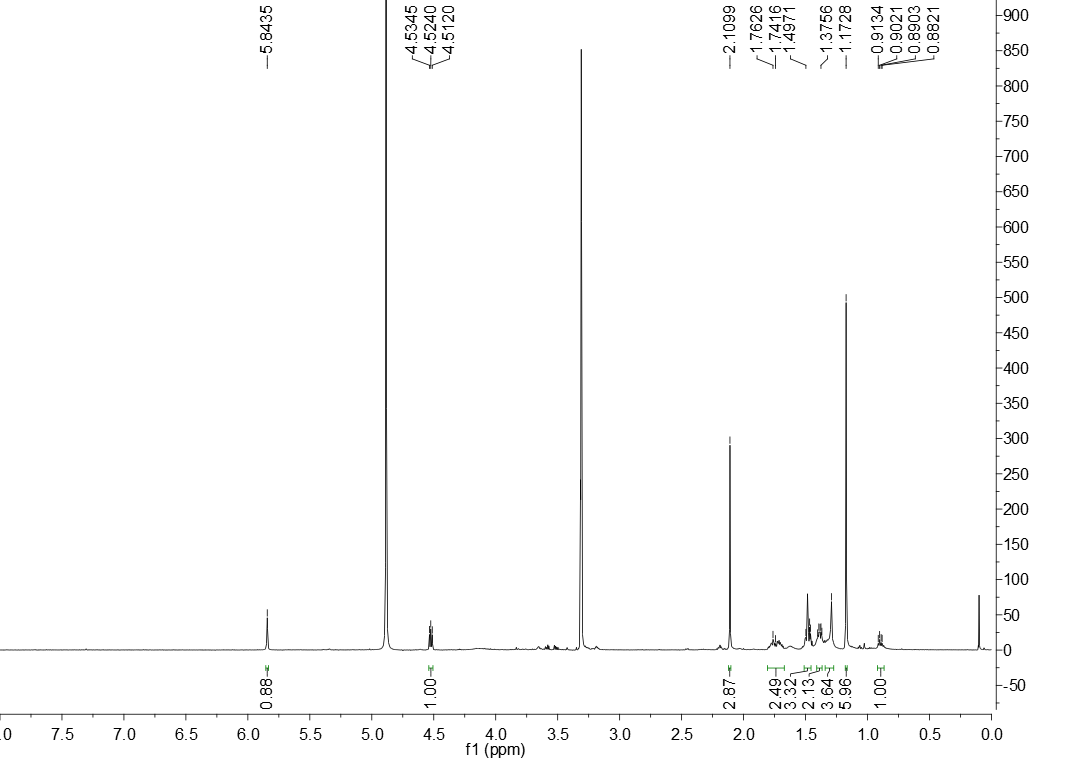


S4-1. ^1^H NMR spectrum of pestaloficin G (4).


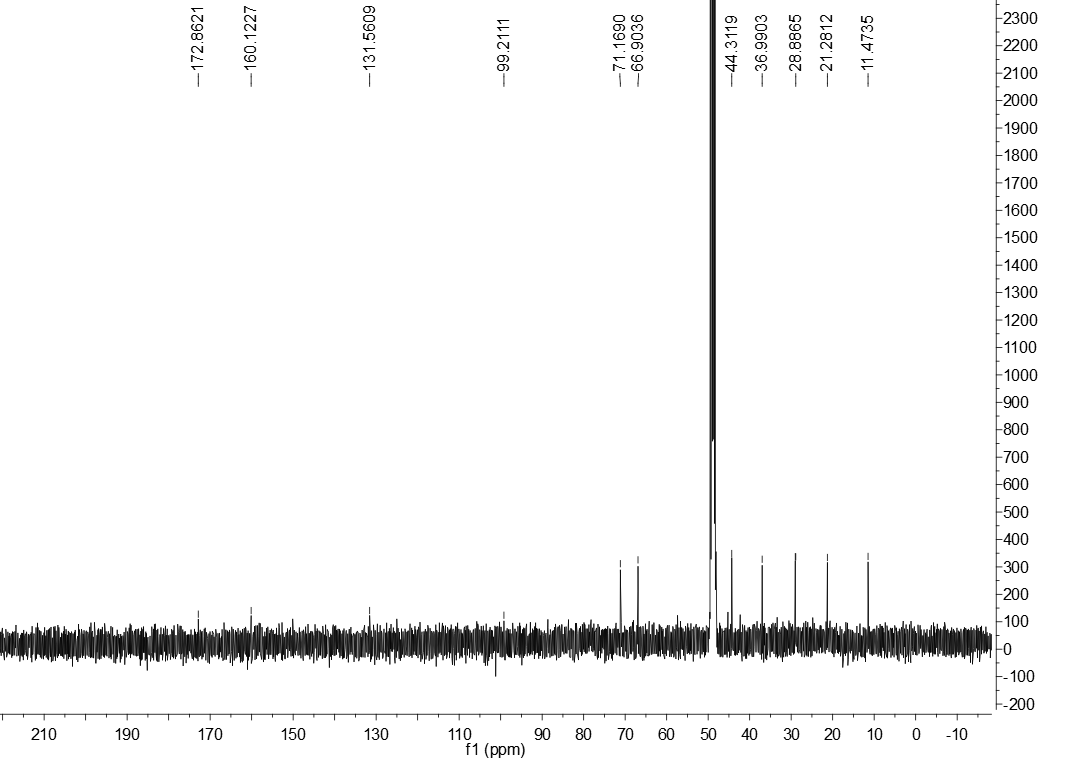


S4-2. ^13^C NMR spectrum of pestaloficin G (4).

S4-3. HRESIMS spectrum of pestaloficin G (4).


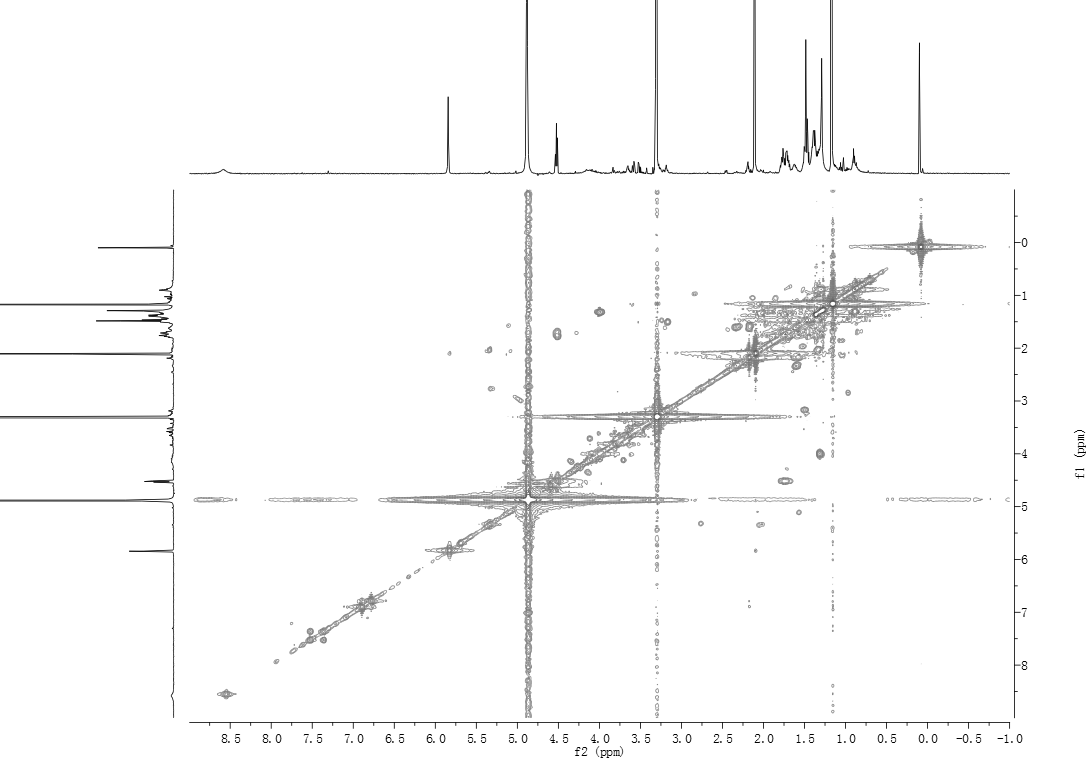


S4-4. ^1^H-^1^H COSY spectrum of pestaloficin G (4).


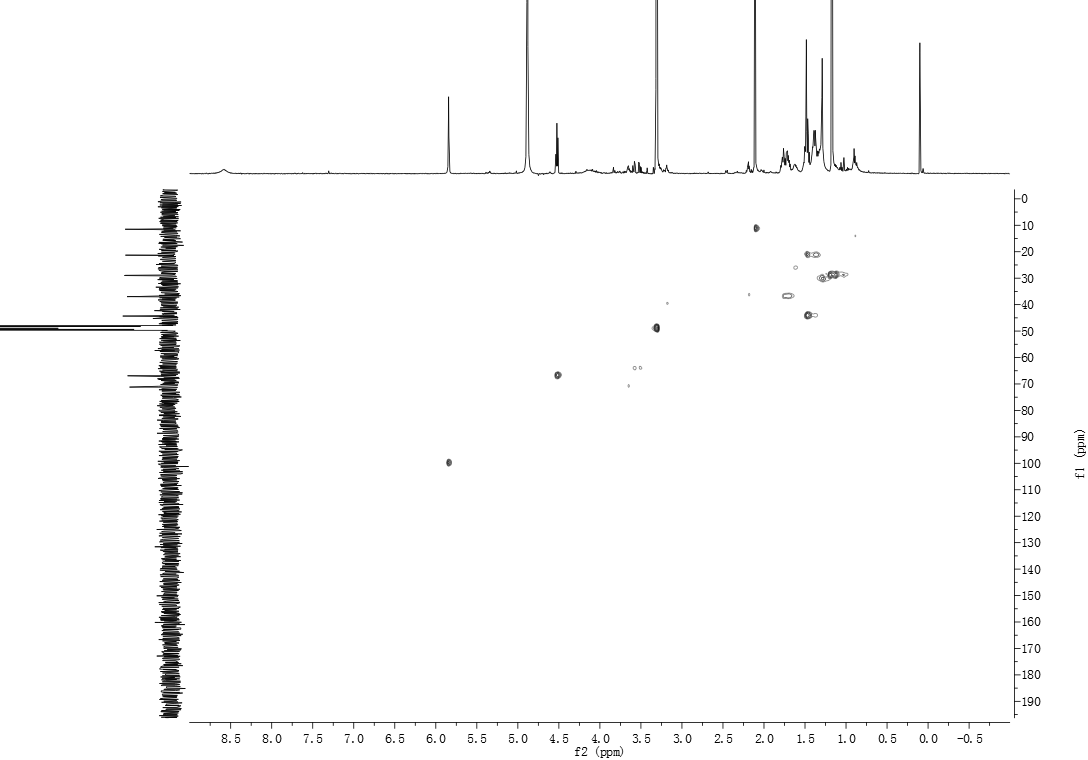


S4-5. HSQC spectrum of pestaloficin G (4).


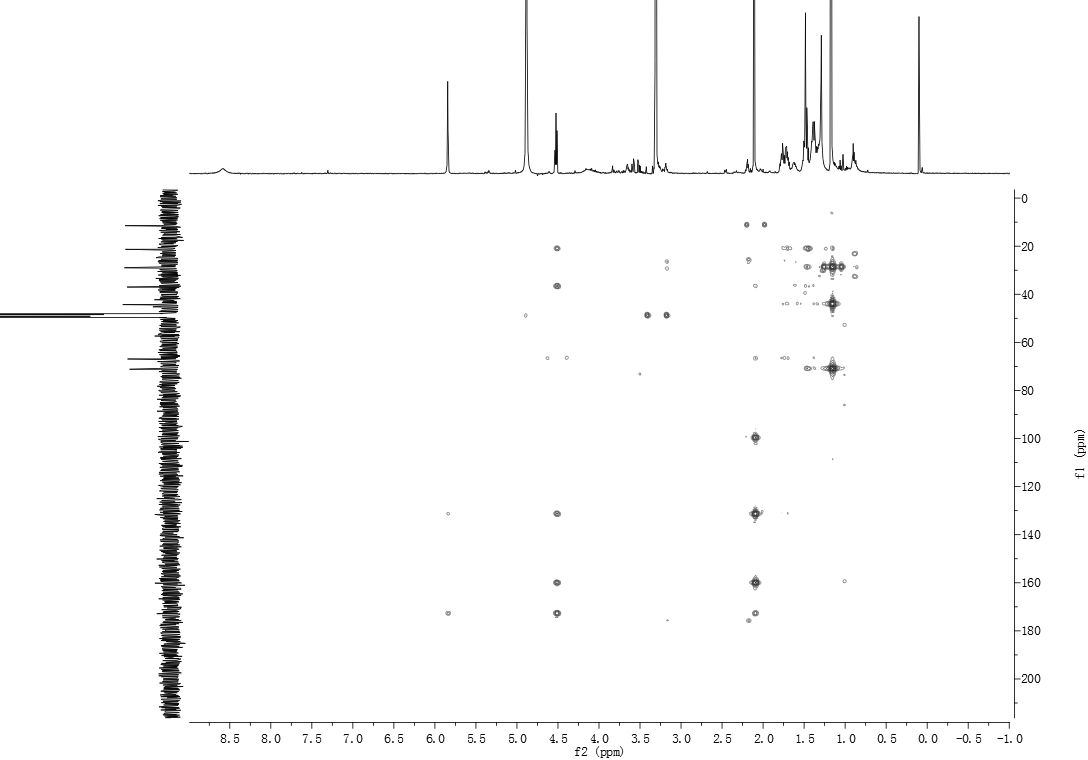


S4-6. HMBC spectrum of pestaloficin G (4).


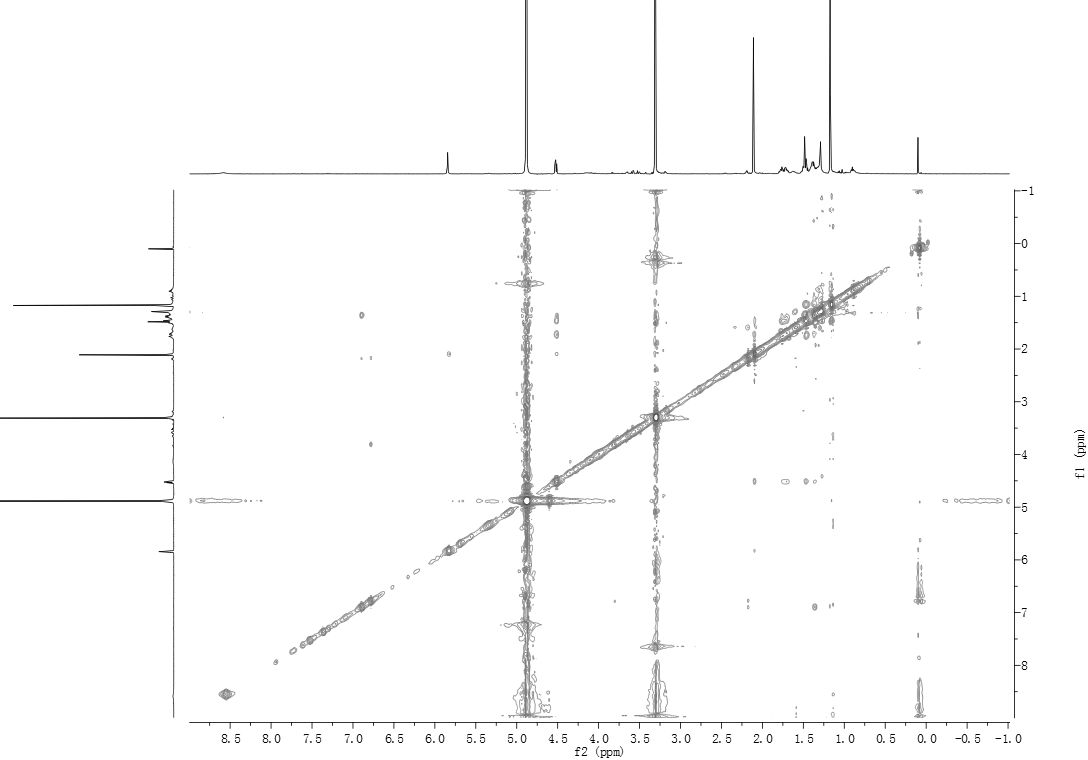


S4-7. NOESY spectrum of pestaloficin G (4).


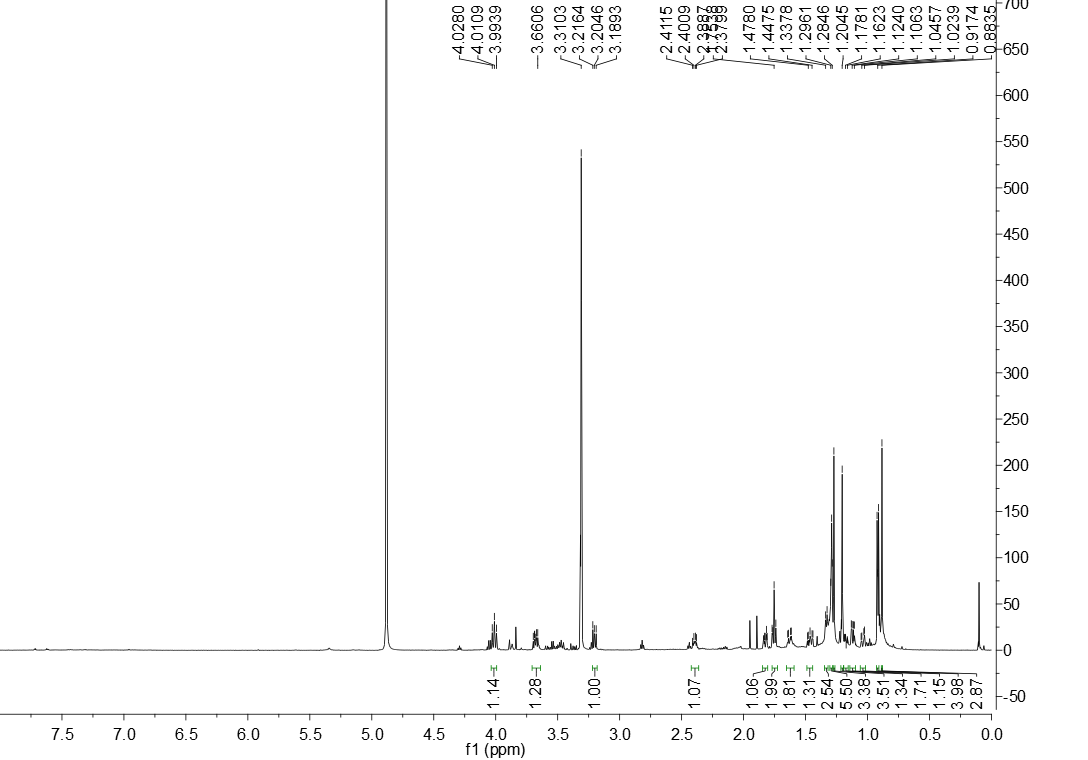


S5-1. ^1^H NMR spectrum of eudesmanetetraiol A (5).


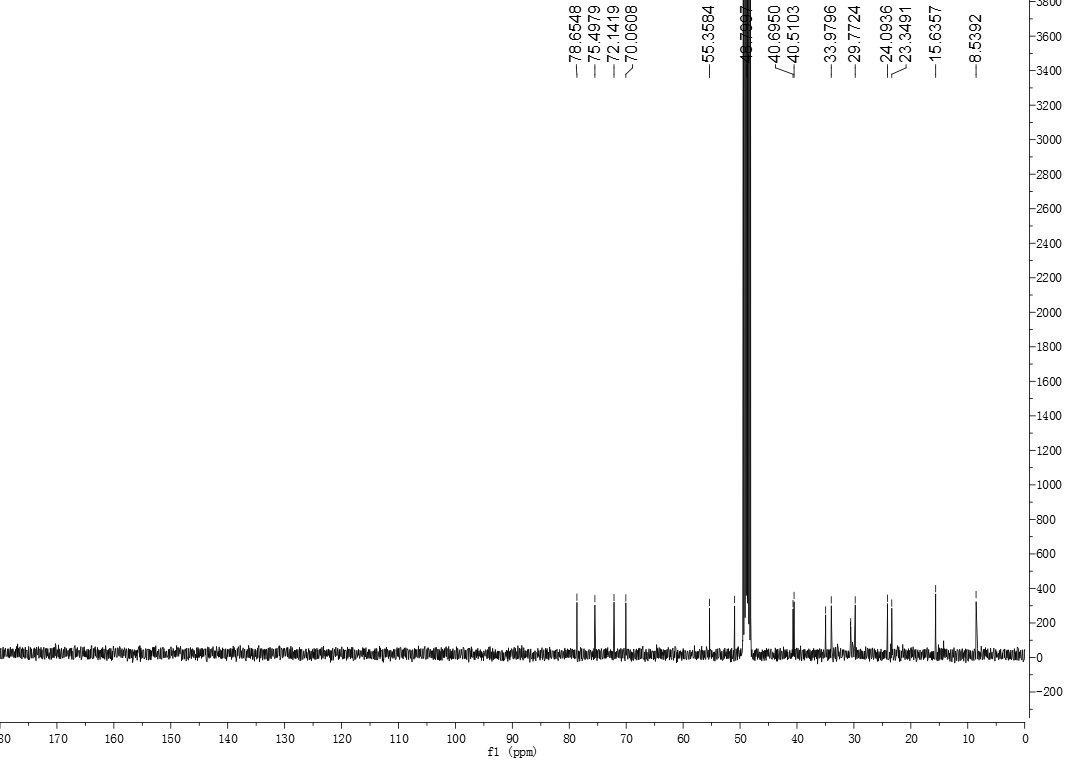


S5-2. ^13^C NMR spectrum of eudesmanetetraiol A (5).

S5-3. HRESIMS spectrum of eudesmanetetraiol A (5).


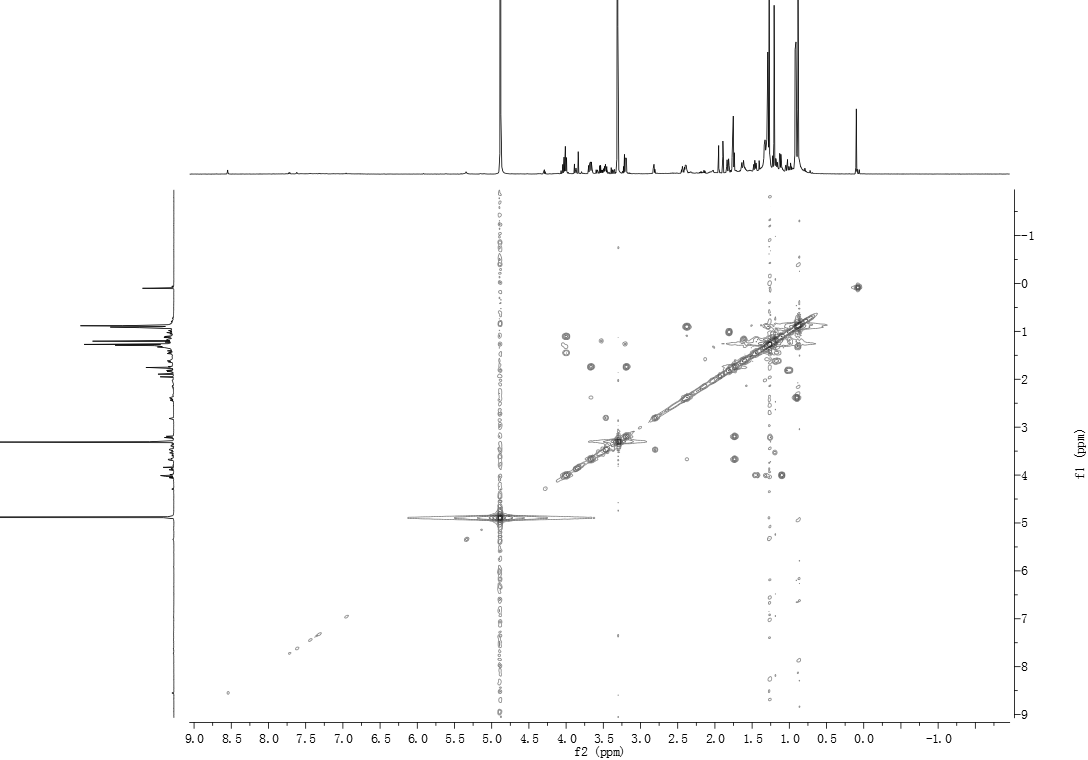


S5-4. ^1^H, ^1^H-COSY spectrum of eudesmanetetraiol A (5).


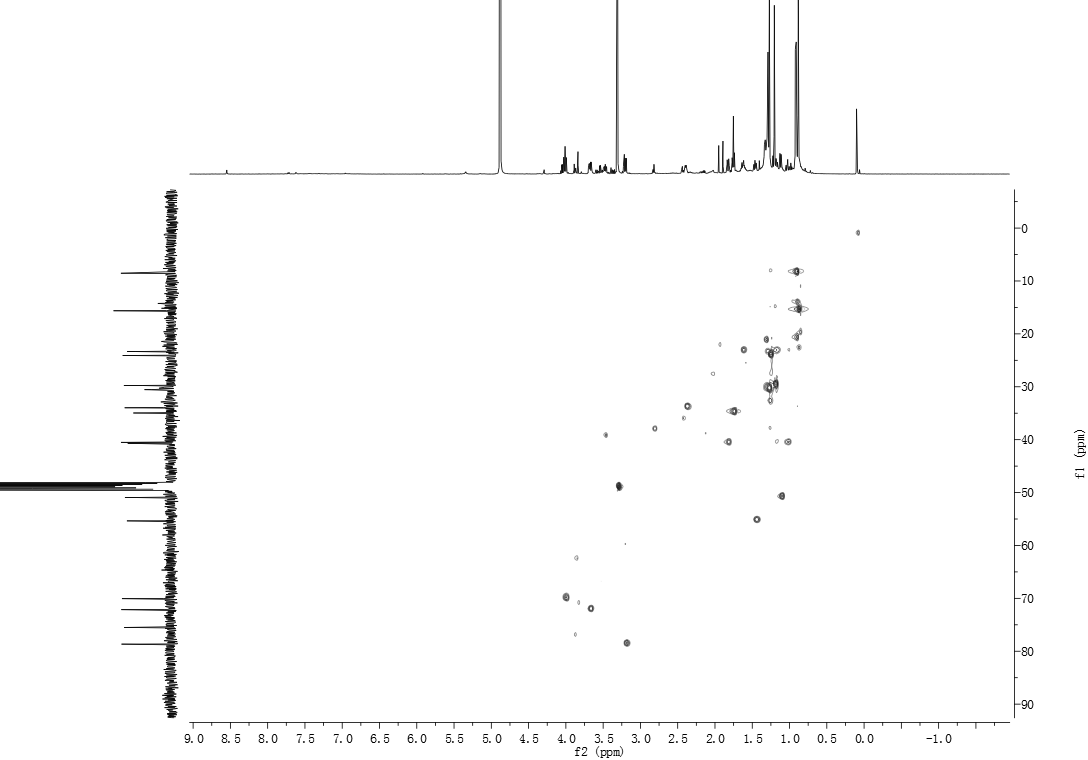


S5-5. HSQC spectrum of eudesmanetetraiol A (5).


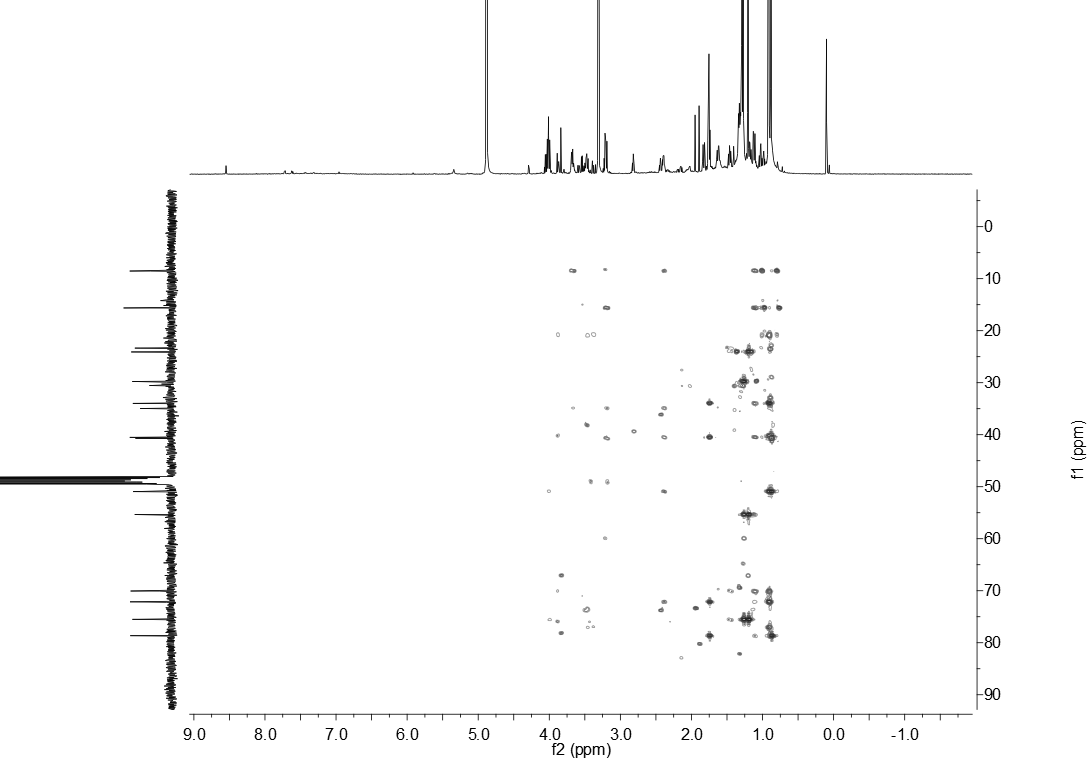


S5-6. HMBC spectrum of eudesmanetetraiol A (5).


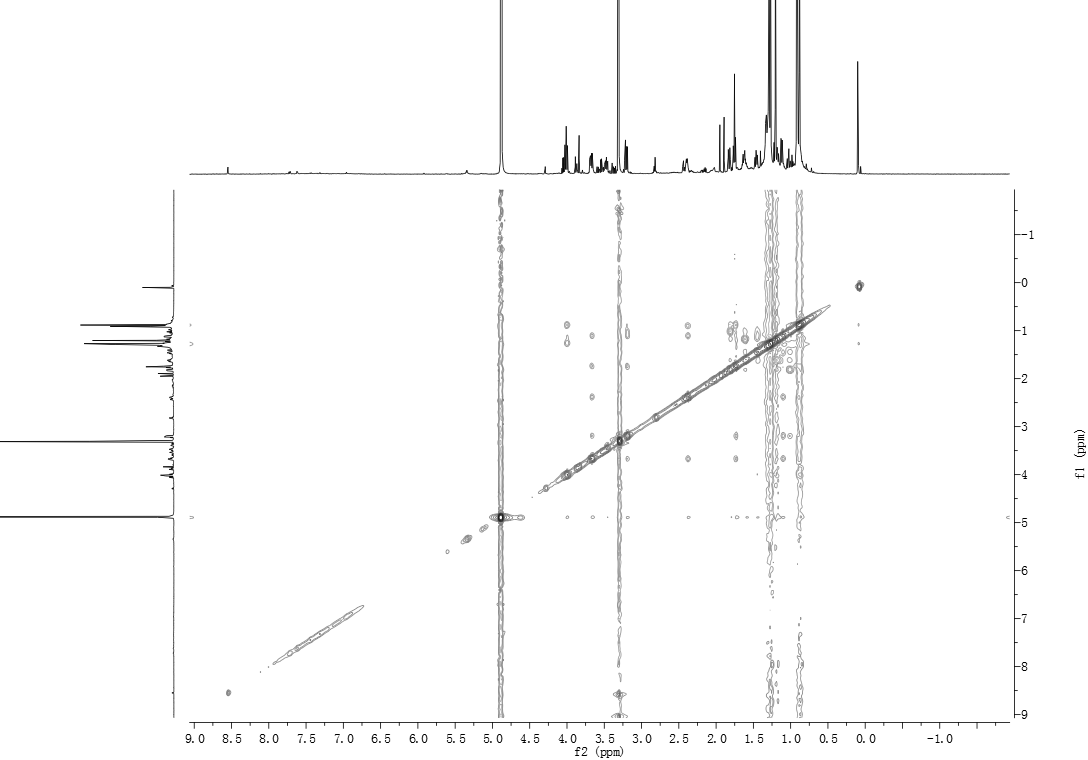


S5-7. NOESY spectrum of eudesmanetetraiol A (5).

**
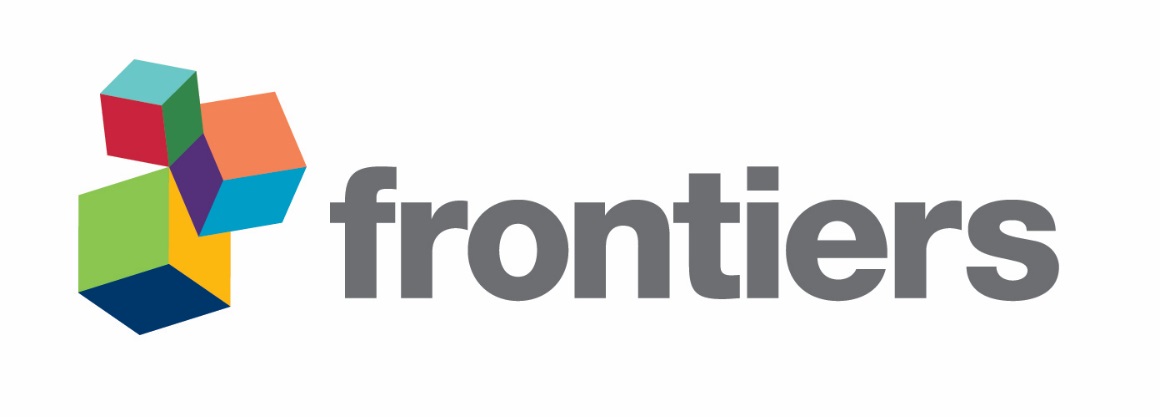
**
